# Increasing the reporting of adverse drug reaction‐related hospitalizations using an ICD‐10‐based identification workflow: A multicentre study from Switzerland

**DOI:** 10.1002/bcp.70564

**Published:** 2026-05-05

**Authors:** Georgia Anita Weber, Sophie‐Charlotte Bartel, Michael Boch, Christoph Rosen, Michael Bodmer, Balthasar Luzius Hug, Thomas Stammschulte, Patrick E. Beeler

**Affiliations:** ^1^ Faculty for Health Sciences and Medicine University of Lucerne Lucerne Switzerland; ^2^ Hospital Pharmacy Lucerne Cantonal Hospital Lucerne Switzerland; ^3^ Hospital Pharmacy, Zug Cantonal Hospital Baar Switzerland; ^4^ Internal Medicine Zug Cantonal Hospital Baar Switzerland; ^5^ Center for Clinical Research Lucerne Cantonal Hospital/University of Lucerne Lucerne Switzerland; ^6^ Internal Medicine Lucerne Cantonal Hospital, University Teaching and Research Hospital Lucerne Switzerland; ^7^ Pharmacovigilance, Safety of Medicines Division, Swissmedic Swiss Agency for Therapeutic Products Berne Switzerland

**Keywords:** adverse drug reaction reporting systems, drug‐related side effects and adverse reactions/epidemiolog, hospitalization/statistics & numerical data, petrospective studies, pharmacoepidemiology/statistics & numerical data, pharmacovigilance, Switzerland

## Abstract

**Background:**

Reporting adverse drug reactions (ADRs) is essential for drug safety. In Switzerland, healthcare professionals are legally required to report serious and unlabelled ADRs, yet under‐reporting remains widespread. We tested a novel method to increase reporting of ADR‐related hospitalizations.

**Methods:**

This retrospective observational study used ADR‐indicative ICD‐10 codes to screen admissions to four Swiss hospitals, identify suspected drugs and send individual case safety reports (ICSRs) of confirmed cases to Swissmedic.

**Results:**

Participating hospitals previously reported ~18 ICSRs annually. During the study period (7/2023–12/2023), 200 ADR‐related hospitalizations were reported following a review of 814 pre‐filtered admissions. Most ICSR data were available in structured format: Median age of patients was 59 (interquartile range [IQR] 42–75); 87 (44%) males; median of two comorbidities (IQR 1–3); the three most frequent ADRs were ‘K52.1 Toxic gastroenteritis and colitis’ (11 [6%]), ‘T42.4 Poisoning by benzodiazepines’ (10 [5%]) and ‘R11 Nausea and vomiting’ (10 [5%]). Discharge reports contained free‐text information on suspected drugs: More than half of ADR‐related hospitalizations were caused by antineoplastics (45 [23%]), psycholeptics (38 [19%]), opioids and other analgesics (34 [17%]). The median time to screen a case was 1 min, 8 min to collect, compile and send data of confirmed cases.

**Conclusion:**

The approach successfully increased reporting of serious ADRs. The time investment for creating ICSRs might soon be rendered obsolete, as most data are available in structured format, and large language models are key to identifying suspected drugs in discharge reports.

**What is already known about this subject**

- Reporting of ADRs is essential for pharmacovigilance and patient safety.
- Although reporting of serious ADRs, and therefore ADRs‐related hospitalizations, is legally mandated in Switzerland, substantial under‐reporting persists.
- A previous retrospective study has demonstrated that ADR‐related hospitalizations can be identified using ICD‐10 codes derived from routinely collected administrative hospital data.

**What this study adds**

- Our study applied ICD‐10 code‐based screening to increase the reporting rate of ADR‐related hospitalizations more than 10‐fold compared to previous years.
- The identified drug classes are consistent with existing literature, supporting the validity of detection strategy.
- The structured workflow provides a foundation for future integration of large language models to further automate case identification and reporting and therefore potentially enhancing efficiency and scalability.

## 1 INTRODUCTION

In Europe, the proportion of hospital admissions due to adverse drug reactions (ADRs) varies from 0.5% to 12.8%.^1^ Similarly, Swiss studies have stated that 2.3%–7.1% of all hospital admissions are caused by ADRs.^2^, ^3^, ^4^ Therefore, ADRs have a severe impact on patient safety and cause increased costs to the healthcare system that could often be avoided.^3^, ^5^, ^6^, ^7^

In Switzerland, the Swiss Agency for Therapeutic Products, Swissmedic, is in charge for the surveillance of the safety of therapeutic products. This monitoring system, known as ‘pharmacovigilance’, consists of the reporting of individual case safety reports (ICSRs), such as ADRs. Healthcare professionals are obliged by Swiss law to report serious or so far unlabelled ADRs. ADRs, resulting in a hospitalization are defined as serious and therefore should be reported. Significant under‐reporting has been recognized in studies, which stated that only 3%–12% of the ADR‐related (re)admissions were reported.^2^, ^8^ In addition, by the Swiss Federal Audit Office (SFAO) identified the potential for more ADR reports by HCPs following an audit of the Swiss pharmacovigilance system.^9^, ^10^, ^11^

As a result, essential information on the safety of therapeutic products may be lost, and therefore, measures to improve patient safety may not be implemented. The aim of this study is to develop an improved, generic approach to detect, verify and report ADRs, leading to hospitalization in Swiss hospitals.

## 2 METHODS

### 2.1 Study design, setting and period

In this cross‐sectional observational study with a retrospective design, we collected data from four Swiss hospitals. Three of these hospitals—located in Luzern, Sursee and Wolhusen—are part of a hospital group in the Canton of Lucerne, whereas the fourth, Zug Cantonal Hospital, is situated in the Canton of Zug. In 2023, these institutions recorded approximately 55 000 inpatient stays.^12^, ^13^ The study period spanned from July to December 2023.

Our study was approved by the responsible ethics committee (BASEC identifier 2023‐00521). Patients with documented refusal of using their health‐related data for research were excluded. This study adhered to the guidelines of Strengthening the Reporting of Observational Studies in Epidemiology (STROBE).^14^

### 2.2 ADR definition and identification

The definition of an ADR used in this study is based on the European Medicines Agency's (EMA) definition: ‘A response to a medicinal product which is noxious and unintended’.^15^ The EMA definition also includes use of medical products at abnormal doses, encompassing abuse, dependence and addiction. Swissmedic states that these also affect the safety profile of a drug and should therefore be reported.^9^

Hospitalizations caused by ADRs are usually diagnosed by healthcare professionals upon admission, and the drug‐related information is documented in the patient's health record. After hospital discharge, all diagnoses are assigned with a specific ICD‐10 code (International Classification of Diseases, World Health Organization [WHO], Geneva Switzerland) by the hospital's internal coding team. This process is standardized by the Swiss ICD‐10 coding rules and is independent of the language region. Each Swiss hospital creates a predefined standard dataset for the cantons and the Federal Statistical Office with ICD‐10 codes and additional data points.^16^

Therefore, ICD‐10 was used as the basis for the identification algorithm because it is the mandatory diagnostic classification system in Swiss hospitals and is available in routinely collected administrative data. In contrast, MedDRA terminology is not coded at the point of care and therefore cannot be used for systematic screening of all admissions. MedDRA coding is applied later by Swissmedic during their pharmacovigilance assessment of submitted reports, which is separate from the identification workflow used in this study.

For the purposes of this study, we define ‘ADR‐related hospitalizations’ as inpatient admissions where an ADR is considered the likely causal factor for the clinical condition leading to hospitalization. To identify such ADR‐related hospitalizations, we applied a previously developed method explained in detail elsewhere.^2^ In short, our approach captures admissions where either (i) the primary diagnosis explicitly denotes a drug‐induced condition (e.g. *T88.6 Anaphylactic shock due to adverse effect of correct drug or medicament properly administered*)^17^ or (ii) the ancillary information or first secondary diagnosis includes specific ICD‐10 codes (*Y57.9 Drug or medicament, unspecified* or *Y59.9 Vaccine or biological substance, unspecified*), indicating that the primary diagnosis was triggered by a medication. A detailed description of the ICD‐10 query logic is provided in the Supporting Information.

### 2.3 Study subjects and variables

The study considered ADR‐related admissions of adult patients. The collected data can be divided into two subsets. First, administrative hospital data generated through standardized processes. Second, manually collected data obtained by reviewing the electronic health record (EHR) of patients admitted due to ADRs. The administrative data contained patient‐related variables such as age, sex and comorbidities,^18^ as well as case‐related variables, such as length of stay (LOS) in the hospital, LOS in the intensive care unit (ICU), primary diagnosis and ancillary information on primary diagnosis and secondary diagnoses. Manually collected data included the time spent reviewing the patient's EHR, ADR‐causing drug, dosage of the ADR‐causing drug, time to onset of ADR and concomitant medications. Furthermore, the location in the EHR where the physicians documented the suspected drug causing the ADR was recorded, for example, in the hospital discharge summary.

Correct documentation of the time to onset of an ADR is particularly emphasized in the study by Bergvall et al. It is stated that imprecise information on time to onset, for example, ambiguity regarding whether the medication was administered before or after the ADR, reduces the quality of the ICSR.^19^

Based on the manually collected data, the ICSRs were electronically sent to Swissmedic via the ‘ElViS’ (Electronic Vigilance System) portal on their website. According to the minimally required criteria, these reports included at least data on (i) the patient concerned, (ii) the reporting person, (iii) the ADR‐causing agent and (iv) the ADR itself. Furthermore, the fully anonymized discharge summary and other relevant information such as drug–drug interactions were sent to Swissmedic.^9^

It is important to note that cases were excluded from the study if they did not fulfil the minimal reporting criteria. Moreover, the completeness and accuracy of the documentation in the EHR depends on the healthcare professionals in charge and cannot be independently verified. In instances of polypharmacy, the identification of the suspected causative drug may be challenging. In addition, the applied ICD‐10‐based identification strategy may not identify all ADRs that may have contributed to an admission. Therefore, potential confounders considered in this study include polypharmacy, comorbidities and possible misclassification due to incomplete or inaccurate documentation in the EHR.

### 2.4 Data processing, sample size and statistics

Prior to this study, ADR reporting in the four participating hospitals followed the Swiss pharmacovigilance framework, in which reporting of ADR‐related hospitalizations is legally mandated but relies on spontaneous submissions by individual healthcare professionals via ElViS. This project introduced an ICD‐10‐based identification and reporting process while continuing to use the existing electronic reporting system.

During data processing, no data cleaning was performed. Some information on additional variables manually collected during the chart review, such as time to onset or dosage, was not available in structured format.

We aimed to identify at least 100 ADR‐related hospitalizations to meet minimum statistical requirements. For feasibility, we limited the review to pre‐filtered admissions from a 6‐month period and planned to stop once 200 confirmed cases were reached. Approximately 23 500 inpatient admissions were considered and ICD‐10 code pre‐filtered in this study: This number reflects the 6‐month data collection period for the Lucerne hospitals and a sample of 20 cases (predefined number) at the Zug hospital requiring a 2‐month data collection period.

The comorbidities of the patients were assigned on the basis of all secondary diagnoses, according to the definitions by Quan et al.^18^ We considered all Elixhauser comorbidities, and, in addition, the Charlson comorbidity *dementia* as suggested by Parameswaran Nair et al.^20^

Categorical variables are displayed as counts and percentages, while continuous variables with non‐normal distribution are displayed as medians and interquartile ranges. For the LOS in the ICU, due to the very low number of hours, we additionally report the mean (with standard deviation). If the variable age is displayed, it is categorized into age groups. Drugs were categorized according to the Anatomical Therapeutic Chemical (ATC) Classification.^21^ For clarity, only the 10 most prevalent drug classes are shown in Table 1. If more than one drug was suspected to be causing an ADR, the case was labelled as ‘two or three drugs suspected’. The ICD‐10 codes representing ADRs were grouped into ICD‐10 code chapters. To improve readability, specific ICD‐10 codes are only listed beneath each chapter if more than one case with this specific code was affected (comprehensive list is available online: Table S7). To assess changes in reporting rates, the numbers of ICSRs submitted to Swissmedic outside of this study from 2016 to 2023 were used for comparison.

**TABLE 1:** Characteristics of patients admitted due to ADRs, stratified by age groups.

Age groups	18–49	50–64	65–79	≥80	Overall
Number of admissions (%)	62 (31)	52 (26)	55 (27.5)	31 (15.5)	200
Sex, male (%)	23 (37.1)	24 (46.2)	27 (49.1)	13 (41.9)	87 (43.5)
Drug classes (%)					
Analgesics without opioids	9 (14.5)	3 (5.8)	2 (3.6)	1 (3.2)	15 (7.5)
Antiepileptics	2 (3.2)	3 (5.8)	2 (3.6)	0 (0.0)	7 (3.5)
Anti‐infectives for systemic use	2 (3.2)	1 (1.9)	5 (9.1)	4 (12.9)	12 (6.0)
Anti‐inflammatory and antirheumatic products	2 (3.2)	2 (3.8)	1 (1.8)	1 (3.2)	6 (3.0)
Antineoplastic agents	4 (6.5)	14 (26.9)	18 (32.7)	2 (6.5)	38 (19.0)
Antithrombotic agents	1 (1.6)	1 (1.9)	3 (5.5)	8 (25.8)	13 (6.5)
Diuretics	1 (1.6)	1 (1.9)	3 (5.5)	2 (6.5)	7 (3.5)
Opioids	8 (12.9)	7 (13.5)	0 (0.0)	2 (6.5)	17 (8.5)
Psychoanaleptics	2 (3.2)	2 (3.8)	0 (0.0)	1 (3.2)	5 (2.5)
Psycholeptics	19 (30.6)	3 (5.8)	4 (7.3)	2 (6.5)	28 (14.0)
Two or three drugs suspected	6 (9.7)	5 (9.6)	0 (0.0)	0 (0.0)	11 (5.5)
Other drug classes	6 (9.7)	10 (19.2)	17 (30.9)	8 (25.8)	41 (20.5)
Concomitant drugs (%)					
No concomitant drug suspected (%)	37 (59.7)	31 (59.6)	46 (83.6)	24 (77.4)	138 (69.0)
Other concomitant drug suspected	24 (38.7)	19 (36.5)	8 (14.5)	7 (22.6)	58 (29.0)
Opioid as concomitant drug suspected	1 (1.6)	2 (3.8)	1 (1.8)	0 (0.0)	4 (2.0)
Comorbidities					
Number of comorbidities	1 [0, 2]	2 [1, 3]	3 [1, 5]	4 [2.50, 5]	2 [1, 3]
Congestive heart failure (%)	0 (0.0)	1 (1.9)	6 (10.9)	8 (25.8)	15 (7.5)
Cardiac arrhythmias (%)	4 (6.5)	2 (3.8)	11 (20.0)	14 (45.2)	31 (15.5)
Valvular disease (%)	1 (1.6)	0 (0.0)	2 (3.6)	8 (25.8)	11 (5.5)
Pulmonary circulation_disorders (%)	0 (0.0)	0 (0.0)	2 (3.6)	1 (3.2)	3 (1.5)
Peripheral vascular disorders (%)	0 (0.0)	1 (1.9)	9 (16.4)	4 (12.9)	14 (7.0)
Hypertension (uncomplicated) (%)	2 (3.2)	5 (9.6)	18 (32.7)	7 (22.6)	32 (16.0)
Hypertension (complicated) 1 (%)	1 (1.6)	2 (3.8)	4 (7.3)	10 (32.3)	17 (8.5)
Paralysis (%)	2 (3.2)	1 (1.9)	2 (3.6)	0 (0.0)	5 (2.5)
Other neurological disorders (%)	5 (8.1)	6 (11.5)	4 (7.3)	1 (3.2)	16 (8.0)
Chronic pulmonary disease (%)	2 (3.2)	2 (3.8)	7 (12.7)	1 (3.2)	12 (6.0)
Diabetes (uncomplicated) (%)	2 (3.2)	3 (5.8)	8 (14.5)	5 (16.1)	18 (9.0)
Diabetes (complicated) (%)	0 (0.0)	2 (3.8)	3 (5.5)	3 (9.7)	8 (4.0)
Hypothyroidism (%)	1 (1.6)	1 (1.9)	4 (7.3)	1 (3.2)	7 (3.5)
Renal failure (%)	4 (6.5)	3 (5.8)	12 (21.8)	12 (38.7)	31 (15.5)
Liver disease (%)	0 (0.0)	2 (3.8)	4 (7.3)	1 (3.2)	7 (3.5)
Peptic ulcer disease excluding bleeding (%)	0 (0.0)	0 (0.0)	0 (0.0)	1 (3.2)	1 (0.5)
Lymphoma (%)	0 (0.0)	1 (1.9)	2 (3.6)	1 (3.2)	4 (2.0)
Metastatic cancer (%)	1 (1.6)	10 (19.2)	9 (16.4)	2 (6.5)	22 (11.0)
Solid tumour without metastasis (%)	0 (0.0)	12 (23.1)	11 (20.0)	4 (12.9)	27 (13.5)
Rheumatoid arthritis or collagen vascular disease (%)	2 (3.2)	0 (0.0)	3 (5.5)	2 (6.5)	7 (3.5)
Coagulopathy (%)	2 (3.2)	2 (3.8)	8 (14.5)	10 (32.3)	22 (11.0)
Obesity (%)	3 (4.8)	3 (5.8)	1 (1.8)	1 (3.2)	8 (4.0)
Weight loss (%)	4 (6.5)	8 (15.4)	9 (16.4)	6 (19.4)	27 (13.5)
Fluid and electrolyte disorders (%)	10 (16.1)	13 (25.0)	18 (32.7)	8 (25.8)	49 (24.5)
Blood loss anaemia (%)	0 (0.0)	0 (0.0)	0 (0.0)	2 (6.5)	2 (1.0)
Deficiency anaemia (%)	1 (1.6)	0 (0.0)	1 (1.8)	1 (3.2)	3 (1.5)
Alcohol abuse (%)	8 (12.9)	4 (7.7)	1 (1.8)	1 (3.2)	14 (7.0)
Drug abuse (%)	13 (21.0)	4 (7.7)	0 (0.0)	0 (0.0)	17 (8.5)
Psychoses (%)	1 (1.6)	3 (5.8)	1 (1.8)	0 (0.0)	5 (2.5)
Depression (%)	8 (12.9)	8 (15.4)	3 (5.5)	2 (6.5)	21 (10.5)
Dementia (%)	0 (0.0)	0 (0.0)	5 (9.1)	1 (3.2)	6 (3.0)
No comorbidities (%)	21 (33.9)	11 (21.2)	7 (12.7)	4 (12.9)	43 (21.5)
Length of stay					
Length of stay in days (median [IQR])	1 [1, 3]	4 [1, 8.25]	6 [3, 13]	5 [3, 9.50]	3.50 [1, 7.25]
Length of stay in the intensive care unit in hours (median [IQR])	0 [0, 17]	0 [0, 0]	0 [0, 0]	0 [0, 0]	0 [0, 8.25]
Length of stay in the intensive care unit in hours (mean [SD])	24.6 (75.5)	11.7 (35.0)	26.1 (80.6)	1.29 (5)	18.1 (62.6)

All described statistical analyses were performed with R, version 4.3.2 (R Foundation for Statistical Computing, Vienna, Austria).

### 2.5 Nomenclature of targets and ligands

Key protein targets and ligands in this article are hyperlinked to corresponding entries in http://www.guidetopharmacology.org and are permanently archived in the Concise Guide to PHARMACOLOGY 2023/24.^22^

## 3 RESULTS

During the study period, approximately 23 500 patients were admitted to the four study hospitals. After ICD‐10‐based pre‐filtering, we screened a total of 814 patient charts until we reached our predefined upper threshold of 200 confirmed ADR‐related hospitalizations. These cases then underwent detailed chart review to extract comprehensive clinical information, which was subsequently submitted to Swissmedic as ICSRs.

Three ADR‐related hospitalizations did not meet the minimum reporting criteria due to insufficient information on the causative drug and were therefore excluded; for example, one case involved an ADR attributed to an ‘Ayurvedic medication’.

### 3.1 Increase in reporting rate

**FIGURE 1:**
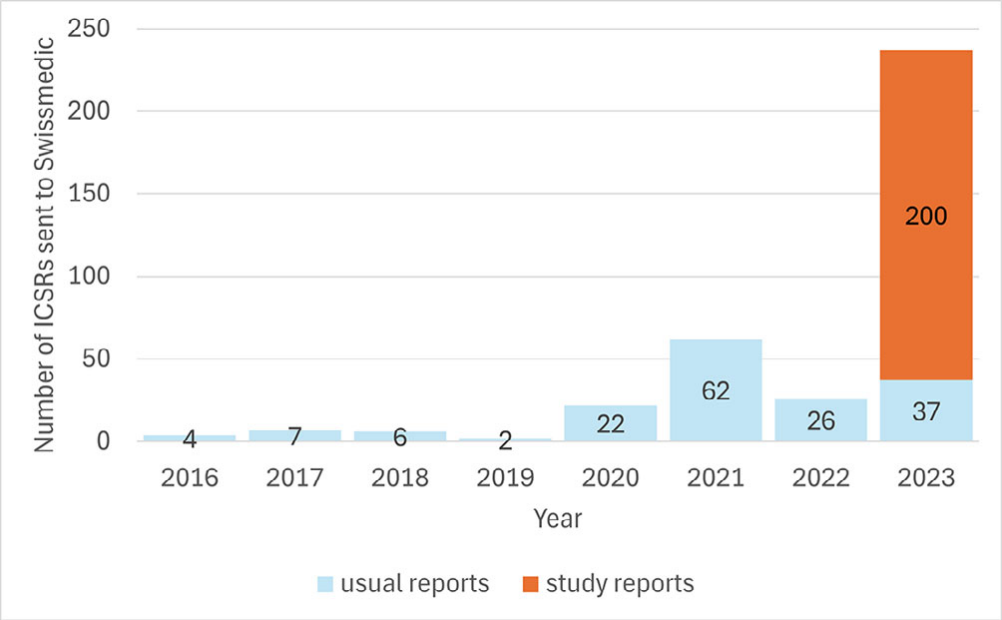
Numbers of spontaneous reports from the four participating hospitals between 2016 and 2023. Blue bars represent the yearly numbers of ICSRs sent using standard spontaneous reporting approaches, and the orange bar represents using our novel ICD‐10‐based approach. Of note, the blue bars included ADR reports in general and not exclusively ADR‐related admissions.

Figure 1 illustrates the increase in the reporting rate. Our approach enabled the identification of substantially more reportable cases than were typically reported in the past. Between 2018 and 2022, the mean number of ADRs reported to Swissmedic across all four hospitals was 18 per year. In the year 2021, which has been documented as a record year, a total of 62 cases were reported. Using our novel approach, we surpassed this number within a short period: We screened admissions to the hospitals Lucerne, Wolhusen and Sursee for 6 months (resulting in 180 ICSRs) and admissions to the hospital in Zug for 2 months (20 ICSRs). Extrapolating from these data, roughly 500 ADR‐related admissions per year can be estimated for the four hospitals in total.

### 3.2 Characteristics of patients admitted for ADRs

The characteristics of the ADR‐related hospital admissions are shown in Table 1. Most of the admitted patients belonged to the age group between 18 and 49 years (*n* = 62, 31%). Male patients accounted for 43.5% (*n* = 87) of the admissions (for a sex‐stratified version, cf. Tables S3–S5). There was a notable difference in the distribution of drug classes across age groups. In the age group of 18–49 years, psycholeptics were the most prevalent drug class (*n* = 19, 30.6%), followed by analgesics without opioids (*n* = 9, 14.5%) and opioids (*n* = 8, 12.9%).The most common comorbidities were fluid and electrolyte disorders (*n* = 49, 24.5%), followed by uncomplicated hypertension (*n* = 32, 16%) and cardiac arrhythmia (*n* = 31, 15.5%). Overall, patients had a median of two comorbidities ([IQR] 1–3), whereas 21.5% (*n* = 43) had none. A higher number of comorbidities was seen in older age groups. Regarding outcomes, the median length of hospital stay was 3.5 days (IQR 1–7.25 days).

### 3.3 ICD‐10 coded diagnoses representing ADRs that led to hospitalization

Table 2 presents the diagnoses associated with ADR‐related hospitalizations, with the corresponding ADR‐specific ICD‐10 codes grouped according to the most prevalent ICD‐10 code chapters. The most prevalent ICD10‐chapter is *S00‐T98 Injury, poisoning and certain other consequences of external causes* (*n* = 64, 32%). Within this chapter, 56 cases (28%) were coded as acute drug poisonings (e.g. benzodiazepines, opioids, antidepressants, antiepileptics), while a smaller proportion represent other ADRs. The second most frequent ICD‐10 chapter was *K00‐K93 Diseases of the digestive system* (*n* = 29, 14.5%). The most prevalent ICD‐10 code is *K52.1 Toxic gastroenteritis and colitis* (*n* = 11, 5.5%).

**TABLE 2:** Overview of the most frequent ICD‐10 coded ADRs that led to hospitalization, grouped by ICD‐10 diagnosis chapters. Only ICD‐10 codes recorded at least twice are shown (complete list is available online: table S7).

ADR ICD‐10 code	Description	Number of admissions (%)
S00–T98	Injury, poisoning and certain other consequences of external causes	64 (32%)
T42.4	Poisoning by benzodiazepines	10
T39.1	Poisoning by 4‐aminophenol derivatives	8
T43.5	Poisoning by other and unspecified antipsychotics and neuroleptics	6
T40.2	Poisoning by other opioids	5
T43.2	Poisoning by other and unspecified antidepressants	5
T38.3	Poisoning by insulin and oral hypoglycaemic (antidiabetic) drugs	4
T42.6	Poisoning by other antiepileptic and sedative‐hypnotic drugs	3
T39.3	Poisoning by other non‐steroidal anti‐inflammatory drugs (NSAID)	3
T43.4	Poisoning by butyrophenone and thioxanthene neuroleptics	2
T88.6	Anaphylactic shock due to adverse effect of correct drug or medicament properly administered	2
T78.3	Angioneurotic oedema	2
T88.7	Unspecified adverse effect of drug or medicament	2
K00–K93	Diseases of the digestive system	29 (14.5%)
K52.1	Toxic gastroenteritis and colitis	11
K25.0	Gastric ulcer acute with haemorrhage	2
K26.0	Duodenal ulcer acute with haemorrhage	2
K52.9	Non‐infective gastroenteritis and colitis, unspecified	2
K71.0	Toxic liver disease with cholestasis	2
R00–R99	Symptoms, signs and abnormal clinical and laboratory findings, not elsewhere classified	23 (11.5%)
R11	Nausea and vomiting	10
R40.0	Somnolence	2
D50–D89	Diseases of the blood and blood‐forming organs and certain disorders involving the immune mechanism	21 (10.5%)
D70.1	Drug‐induced agranulocytosis and neutropenia	8
D68.3	Haemorrhagic disorder due to circulating anticoagulants	6
D76.4	Cytokine release syndrome	3
F01–F99	Mental and behavioural disorders	14 (7%)
F13.0	Mental and behavioural disorders due to use of sedatives or hypnotics—acute intoxication	3
F11.0	Mental and behavioural disorders due to use of opioids—acute intoxication	2
F11.4	Mental and behavioural disorders due to use of opioids—withdrawal state with delirium	2
F13.1	Mental and behavioural disorders due to use of sedatives or hypnotics—harmful use	2
E00–E90	Endocrine, nutritional and metabolic diseases	10 (5%)
E87.1	Hypo‐osmolality and hyponatraemia	4
I00–I99	Diseases of the circulatory system	8 (4%)
I95.2	Hypotension due to drugs	4
J00‐J99	Diseases of the respiratory system	8 (4%)
J70.2	Acute drug‐induced interstitial lung disorders	6
L00–L99	Diseases of the skin and subcutaneous tissue	8 (4%)
L27.0	Generalized skin eruption due to drugs and medicaments	4
G00–G99	Diseases of the nervous system	6 (3%)
G24.0	Drug‐induced dystonia	2
A00–B99	Certain infectious and parasitic diseases	3 (1.5%)
A04.7	Enterocolitis due to *Clostridium difficile*	3
N00–N99	Diseases of the genitourinary system	2 (1%)
	Other ICD‐10 coded ADRs	4 (2%)

### 3.4 Drugs causing ADRs

The most frequent suspected drug classes associated with ADR‐related hospitalizations (Table 3) were antineoplastic agents (*n* = 45, in 22.5% of hospitalizations), psycholeptics (*n* = 38, 19.0%), opioids (*n* = 18, 9.0%), analgesics without opioids (*n* = 16, 8.0%), antithrombotic agents (*n* = 13, 6.5%) and anti‐infectives for systemic use (*n* = 12, 6.0%).

**TABLE 3:** Drug classes suspected in cases of ADR‐related hospitalizations (total number of suspected *n* = 216). If multiple drugs were suspected to have contributed to an ADR, each suspected drug was counted as a separate entry.

ATC code	Description of the ATC code level	Number of suspected drugs (% of total [*n* = 216])
L01	Antineoplastic agents	45 (20.8%)
N05	Psycholeptics	38 (17.6%)
N02A	Opioids	18 (8.3%)
N02	Analgesics without opioids	16 (7.4%)
B01	Antithrombotic agents	13 (6.0%)
J01	Anti‐infectives for systemic use	12 (5.6%)
M01	Anti‐inflammatory and antirheumatic products	9 (4.2%)
N06	Psychoanaleptics	9 (4.2%)
N03	Antiepileptics	8 (3.7%)
C03	Diuretics	7 (3.2%)
A10	Drugs used in diabetes	5 (2.3%)
N04	Anti‐Parkinson drugs	5 (2.3%)
C07	Beta‐blocking agents	3 (1.4%)
L04	Immunosuppressants	3 (1.4%)
C01	Cardiac therapy	2 (0.9%)
H02	Corticosteroids for systemic use	2 (0.9%)
M03	Muscle relaxants	2 (0.9%)
N07	Other nervous system drugs	2 (0.9%)
A02	Drugs for acid‐related disorders	1 (0.5%)
A03	Drugs for functional gastrointestinal disorders	1 (0.5%)
B05	Blood substitutes and perfusion solutions	1 (0.5%)
C08	Calcium channel blockers	1 (0.5%)
C09	Agents acting on the renin–angiotensin system	1 (0.5%)
D01	Antifungals for dermatological use	1 (0.5%)
G02	Other gynaecologicals	1 (0.5%)
H03	Thyroid therapy	1 (0.5%)
J04	Antimycobacterials	1 (0.5%)
J05	Antivirals for systemic use	1 (0.5%)
L03	Immunostimulants	1 (0.5%)
M04	Antigout preparations	1 (0.5%)
N01	Anaesthetics	1 (0.5%)
P01	Antiprotozoals	1 (0.5%)
S01	Ophthalmologicals	1 (0.5%)
V08	Contrast media	1 (0.5%)
P02^a^	Anthelmintics	1 (0.5%)

^a^ Only used in veterinary medicine.

**FIGURE 2:**
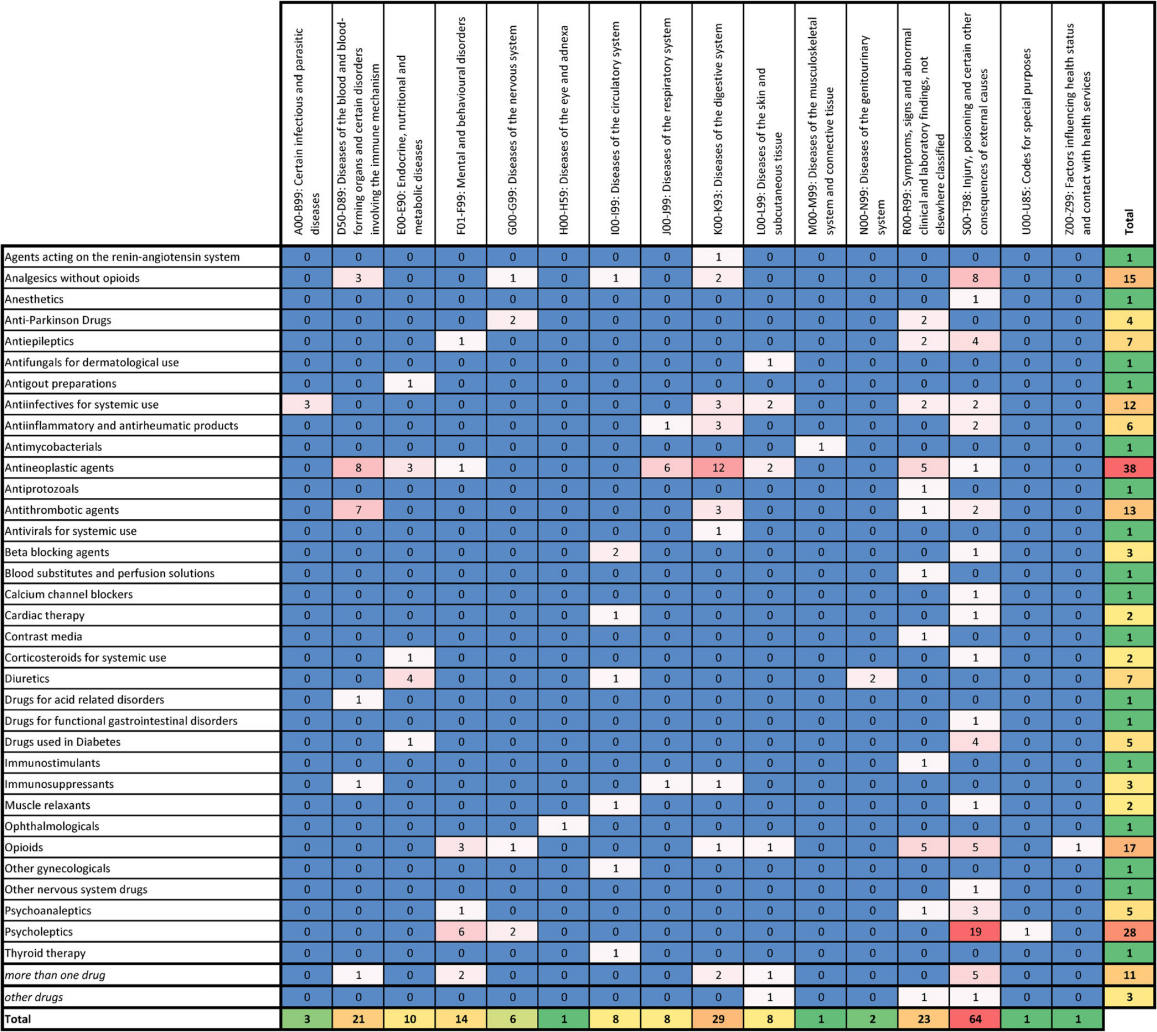
Frequency overview of drug classes leading to ADR‐related hospitalizations. ADRs are grouped into ICD‐10 code chapters. Detailed data and additional information are available in table S6.

At the first ATC level (cf. Table S1), nervous system drugs represented the largest group (*n* = 97, 48.5%), followed by antineoplastic and immunomodulating agents (*n* = 49, 24.5%). Table S2 shows that the most often suspected drug for ADR‐related hospital admissions was alprazolam (*n* = 9, 4.5%), followed by metamizole, pembrolizumab and quetiapine (each *n* = 8, 4.0%). Figure 2 illustrates the identified drugs and their ADRs.

### 3.5 Time needed to screen, collect data of confirmed cases and report

For excluded cases (*n* = 614), the median screening time was 1.0 min (mean: 2.2 min). For the first 80 confirmed ADRs, the median time required for chart review and data collection was 9.4 min (mean: 9.4 min). For the final 34 ICSRs compiled and submitted by a trained hospital pharmacist who regularly submits pharmacovigilance reports, the median time for chart review and data collection was 7.0 min (mean: 7.6 min). The latter included anonymizing the discharge report and retrieving and anonymizing prior prescriptions. The median time to submit the report via ElViS was 1.0 min (mean: 1.1 min), resulting in a total median time of 8.0 min (mean: 8.7 min) per confirmed case.

## 4 DISCUSSION

The present study demonstrates a novel approach to improve the issue of under‐reporting of ADRs. Applying this approach across four Swiss hospitals, we demonstrated a substantial increase in detection and reporting of serious ADRs. Antineoplastic agents were the most common drug class associated with ADR‐related hospitalizations.

### 4.1 Strengths

This multicentre pilot project collected data from four different Swiss hospitals, located in two cantons, belonging to two different hospital groups with distinct IT processes and using different hospital software products, not only for clinical documentation but also for database queries and data extraction. It is worth mentioning that these circumstances did not pose obstacles to the identification of ADRs or the data collecting process during this study due to the standardized nature of the ICD‐10 coding rules equally applied in all Swiss hospitals.

### 4.2 Limitations

This study has several limitations. Firstly, the completeness and accuracy of the documentation of the ADR in the EHR vary, which might have caused bias. Therefore, three cases could not be reported to Swissmedic, as the minimum required criteria for a spontaneous report were not fulfilled. Secondly, it was not always possible to clearly identify the suspected causative drug, especially in cases of polypharmacy. This reflects a general challenge to identify the causative drug, particularly in multimorbid older adults with multiple medications, as already described previously in literature.^23^, ^24^ This may result in under‐recognition compared to younger patients, who are less likely suffering from multimorbidity or polypharmacy. Thirdly, although our approach clearly increased the reporting rate, we may still have missed certain cases. Compared to other approaches such as trigger tool application combined with detailed chart review approaches, our ICD‐10 code‐based strategy may have a lower sensitivity as stated in the study of Wickham et al.^25^ However, higher sensitivity relies on labour‐intensive chart reviews and expert consensus, which is costly and may limit feasibility in routine surveillance. Additionally, these methods may identify ADRs that cannot be declared as clear ADRs but more as potential ADRs.^25^, ^26^, ^27^, ^28^ Finally, this study was conducted within the context of a regional setting; therefore, generalizability of the findings is limited on a national or international level.

### 4.3 Interpretation of primary results

The study population was heterogeneous, with a median age of 59 years and a substantial proportion of patients in the 18–49‐year age group. The most frequent ICD‐10 chapter associated with ADR‐related hospitalizations was *S00‐T98 Injury, poisoning and certain other consequences of external causes*. Although this chapter also contains non‐poisoning ADRs, the majority of cases within this group reflected acute drug intoxications, consistent with our definition of ADRs, which includes overdose and misuse. The second most frequent ICD‐10 chapter was *K00‐K93 Diseases of the digestive system*. This finding is consistent with the results of previous reports, which indicated that ADRs involving the digestive system are among the most prevalent manifestations of ADRs leading to admission.^2^, ^29^ The higher prevalence of women affected by ADRs has also been reported in other studies.^30^, ^31^

### 4.4 Increased reporting rate

Compared to the mean number of ICSRs in the previous years in the four participating hospitals, an 11‐fold increase in the reporting rate was demonstrated using our novel approach. Extrapolating our number of reports sent to Swissmedic to an entire year could potentially result in a 30‐fold increase, further highlighting the well‐known issue of ADR under‐reporting.^2^ Tadge et al and De Vries et al state that time constraints and the high workload of clinically working physicians, nurses and pharmacists are among the main reasons for under‐reporting.^32^, ^33^ In contrast to the current Swiss pharmacovigilance system, which depends on ICSRs reported by healthcare professionals, our approach establishes a structured workflow. ADR‐related hospitalizations are systemically identified through ICD‐10‐based screening of routinely collected administrative data. Once a case is confirmed, the verification and reporting process is centrally performed by a trained pharmacovigilance professional (e.g. a trained hospital pharmacist). Therefore, the responsibility for case verification and submission of the ICSR to Swissmedic is transferred from frontline healthcare professionals to designated pharmacovigilance specialists.

Other approaches—such as those described by Grossmann et al, Thevelin et al and Classen et al—use detailed chart reviews including trigger tools and multidisciplinary adjudication to identify a broader range of potential ADRs but are resource intensive and not feasible for continuous surveillance.^26^, ^27^, ^28^ In order to address known limitations of ICD‐10‐based detection,^25^ we expanded the search beyond primary diagnoses and included additional relevant codes from secondary diagnoses and ICD‐10‐coded ancillary information, as previously suggested.^2^ The present approach combines targeted screenings and chart reviews based on optimized pre‐filtering, thereby trying to offer a compromise between completeness and practicality. Notably, the time required to identify an ADR using our approach is markedly shorter compared to Thevelin et al, who reported a mean of 23 min per case for individual assessment plus an additional 13 min for consensus discussion.^26^

### 4.5 Drug classes

In comparison to previous studies, the present study identified ADR‐causing drugs and organized them into the ATC Classification.^2^, ^21^ This approach enables a more detailed depiction of the pharmacological groups most frequently involved in ADRs.

The analysis identified nervous system drugs as the most common ADR‐related drugs leading to hospital admissions. Within the more specific ATC subgroups, antineoplastic agents ranked highest, followed by psycholeptics, analgesics (of which more than half were opioids), antithrombotic agents and anti‐infectives for systemic use.

This ranking partially aligns with findings of previous studies in this field. For instance, Dubrall et al stated nervous system drugs to be the leading cause of spontaneous ADR reports in Germany. As Dubrall et al previously demonstrated, four of five subgroups—antithrombotic agents, antibacterials for systemic use, psycholeptics and antineoplastic agents—are also represented in the top five subgroups in our study, which suggests that certain drug groups are prone to triggering ADRs.^30^ In another Swiss study, Banholzer et al similarly found antineoplastic and immunomodulating agents as prevalent, though antithrombotics ranked higher in their older study population.^8^ Age might explain this difference, since our median age was 59 *vs*. 67 years in the study by Banholzer et al. This is consistent with Budnitz et al and Kauppila et al, who also observed antithrombotics as the main causative drugs of ADRs in older cohorts.^29^, ^34^ Nevertheless, our definition of an ADR includes misuse and overdose, which may also explain the high prevalence of nervous system drugs, particularly opioids, observed in younger demographics. This matches findings of the Toxicology Investigators Consortium 2023 Annual Report of the American College of Medical Toxicology, which reported that among hospitalized patients requiring toxicology consultation opioids were among the most frequently implicated drug classes in ADRs leading to hospitalization, followed by non‐opioid analgesics and antidepressants, particularly in adults aged 19–65 years.^35^ Similarly, Mirošević et al found that ADRs due to nervous system drugs occurred most frequently in the age group of 41–64 years, which partially matches the demographics of our patient population.^36^

The relevance of antineoplastic agents is further reflected in recent approvals by Swissmedic, with most novel drugs in 2023–2024 belonging to the oncology and hematologic neoplasm category,^37^, ^38^ potentially indicating their increasing importance going forward.

Opioids were the third most common drug class associated with ADR‐related hospitalization. This is notable given the high rates of opioid use and poisonings in Switzerland from 2000 and 2019.^39^ Chiapinni et al also observed an increase in ADRs caused by opioids in Europe and the United States, especially in the age group from 25 to 44 years.^40^

### 4.6 Implications

This project aimed to develop and demonstrate a novel approach applicable to all Swiss hospitals to improve hospitals' reporting of serious ADRs, thereby contributing to enhanced drug safety. Given the nationwide standardization of ICD‐10 coding in Switzerland, independent of language region, the approach is structurally suited for implementation across hospitals at a national level. In three of the participating hospitals, this novel approach has now partially been implemented to sustain an increased reporting rate, which is considered a pharmacovigilance quality indicator. To further support long‐term implementation, assigning a pharmacovigilance‐responsible professional in each hospital may help embed the workflow and reduce clinicians' burden of ADR reporting. Complementing this, providing physicians with an annual pharmacist‐led feedback summary of ADRs identified and submitted through the ICD‐10‐based concept could further strengthen awareness, reinforce feedback loops and foster sustained engagement in pharmacovigilance activities. Findings from a recent nationwide survey of Swiss physicians and pharmacists corroborate the need for such measures, since time burden, unfamiliarity with the reporting system and uncertainty about reporting criteria remain common obstacles, and many respondents request clearer guidance and training.^41^

This study focused exclusively on ADRs leading to hospitalization, as these are classified as serious.^9^ In future research, ADRs occurring during hospitalization could be investigated using a similar ICD‐10‐based strategy. However, not all such ADRs meet the criteria for seriousness or clinical importance, which may limit their prioritization in routine surveillance. It is also important to note that ADRs that do not result in hospitalization may still impact on patient safety. Further research is required to explore the burden of these ADRs, as stated by Bouvy et al.^1^ Given the generalizability of our approach, it may also be adapted for populations not included in this study—for example, children and adolescents.

At present, the ADR‐causing drug and some additional information had to be manually extracted from patients' EHRs. Reporting to Swissmedic was also carried out manually. Nevertheless, a substantial portion of patient and case‐related data can already be automatically extracted by the coding teams, highlighting the feasibility of partial automation. As for the future, large language models hold the potential to automate the extraction of manually collected data and subsequent reporting of ADRs to regulatory authorities.^32^ This could not only help to reduce the workload for healthcare professionals but also render chart reviewing obsolete.

## 5 CONCLUSIONS

Using a novel ICD‐10‐based screening approach, we substantially increased the number of reports of serious ADRs across four Swiss hospitals. This simple method revealed the untapped potential of routine hospital data for pharmacovigilance and demonstrated that structured hospital data and targeted screening can transform ADR surveillance, enabling scalable reporting without burdening clinicians. By leveraging existing ICD‐10 codes and standardized data formats, our approach offers a feasible path to nationwide improvement in ADR reporting. The findings highlight that specific drug classes—particularly antineoplastic agents, psycholeptics, opioids, other analgesics and antithrombotics—are disproportionately associated with serious ADRs leading to admission. As most reporting‐relevant information is already available in structured form, automation—such as through large language models—represents a promising next step. Overall, this scalable strategy enhances pharmacovigilance and underscores the value of hospital data in improving patient safety.

## AUTHOR CONTRIBUTIONS

**Georgia Anita Weber:** Investigation; data curation; formal analysis; visualization; writing—original draft. **Sophie‐Charlotte Bartel:** Investigation; data curation; writing—review and editing. **Michael Boch:** Investigation; writing—review and editing. **Christoph Rosen:** Investigation; writing—review and editing. **Michael Bodmer:** Resources; writing—review and editing. **Balthasar Luzius Hug:** Resources; writing—review and editing. **Thomas Stammschulte:** Methodology; resources; writing—review and editing. **Patrick E. Beeler:** Conceptualization; project administration; supervision; methodology; data curation; visualization; writing—review and editing.

## CONFLICT OF INTEREST STATEMENT

Patrick E. Beeler has received fees and funding from AstraZeneca outside the submitted work. The remaining authors have no competing interests to declare.

## Supporting information


**Data S1.** Supporting Information.

**Table S1:** Most frequently suspected drug classes grouped by the first ATC‐level, total *n* = 216. If multiple drugs were suspected to have contributed to an ADR‐related hospitalization, each suspected drug was counted as a separate entry.
**Table S2:** Drug classes suspected in cases of ADR‐related hospitalizations (total suspected *n* = 216). If multiple drugs were suspected to have contributed to an ADR, each suspected drug was counted as a separate entry.
**Table S3:** Characteristics of admissions, stratified by sex with focus on comorbidities.
**Table S4:** Characteristics of admissions, stratified by sex with focus on drug classes.
**Table S5:** Characteristics of admissions, stratified by sex with focus on ICD‐10 chapters of diagnoses for ADRs.
**Table S6:** Characteristics of admissions, stratified by ICD‐10 code chapters for ADRs.
**Table S7:** Overview of all ICD‐10 coded ADRs that led to hospitalization, grouped by ICD‐10 code chapters.

## Data Availability

The Supporting Information includes the supplementary tables, the full ICD‐10 query logic used to identify admissions for screening and a detailed anonymized dataset of confirmed ADR‐related hospitalizations. In addition to the variables reported in the main manuscript, the anonymized dataset contains further information such as drug administration route, dosage, time to onset in both exact and categorized forms and the documentation source within the EHR. All materials are available in the supplementary files accompanying this publication.
