# Supplementary material for: Increasing the reporting of adverse drug reaction‐related hospitalizations using an ICD‐10‐based identification workflow: A multicentre study from Switzerland
Source: Br J Clin Pharmacol. 2026 May 5;92(8):2937–48. doi: 10.1002/bcp.70564 (PMC13420890; doi:10.1002/bcp.70564)
Supplement: Supplementary file 2 — Table S1: Most frequently suspected drug classes grouped by the first ATC‐level, total n = 216. If multiple drugs were suspected to have contributed to an ADR‐related hospitalization, each suspected drug was counted as a separate entry. Table S2: Drug classes suspected in cases of ADR‐related hospitalizations (total suspected n = 216). If multiple drugs were suspected to have contributed to an ADR, each suspected drug was counted as a separate entry. Table S3: Characteristics of admissions, stratified by sex with focus on comorbidities. Table S4: Characteristics of admissions, stratified by sex with focus on drug classes. Table S5: Characteristics of admissions, stratified by sex with focus on ICD‐10 chapters of diagnoses for ADRs. Table S6: Characteristics of admissions, stratified by ICD‐10 code chapters for ADRs. Table S7: Overview of all ICD‐10 coded ADRs that led to hospitalization, grouped by ICD‐10 code chapters. [file BCP-92-2937-s002.docx]

## Supplementary tables

Supplementary Table 1: Most frequently suspected drug classes grouped by the first ATC-level, total n = 216. If multiple drugs were suspected to have contributed to an ADR-related hospitalization, each suspected drug was counted as a separate entry.

| **ATC code** | **Description of the ATC code** | **Number of suspected drugs (% of total [n=216])** |
| --- | --- | --- |
| N | Nervous system | 97 (44.9%) |
| L | Antineoplastic and immunomodulating agents | 49 (22.7%) |
| B | Blood and blood forming organs | 14 (6.5%) |
| C | Cardiovascular system | 14 (6.5%) |
| J | Antiinfectives for systemic use | 14 (6.5%) |
| M | Musculo-skeletal system | 12 (5.6%) |
| A | Alimentary tract and metabolism | 7 (3.2%) |
| H | Systemic hormonal preparations, excluding sex hormones and insulins | 3 (1.4%) |
| D | Dermatologicals | 1 (0.5%) |
| G | Genito-urinary system and sex hormones | 1 (0.5%) |
| P | Antiparasitic products, insecticides and repellents | 1 (0.5%) |
| S | Sensory organs | 1 (0.5%) |
| V | Various | 1 (0.5%) |
| P^1^ | Antiparasitic products, insecticides and repellents | 1 (0.5%) |

*^1^ i.e. levamisole, which is only used in veterinary medicine*

Supplementary Table 2: Drug classes suspected in cases of ADR-related hospitalizations (total suspected n=216). If multiple drugs were suspected to have contributed to an ADR, each suspected drug was counted as a separate entry.

| **ATC_Code** | **Drug** | **Number of suspected drugs (% of total [n=216])** |
| --- | --- | --- |
| N05BA12 | Alprazolam | 9 (4.2%) |
| L01FF02 | Pembrolizumab | 8 (3.7%) |
| N02BB02 | Metamizole sodium | 8 (3.7%) |
| N05AH04 | Quetiapine | 8 (3.7%) |
| J01CR02 | Amoxicillin and beta-lactamase-inhibitors | 7 (3.2%) |
| N02BE01 | Paracetamol | 7 (3.2%) |
| N05BA06 | Lorazepam | 7 (3.2%) |
| N02AA05 | Oxycodone | 6 (2.8%) |
| M01AE01 | Ibuprofen | 5 (2.3%) |
| N02AA01 | Morphine | 5 (2.3%) |
| B01AF02 | Apixaban | 4 (1.9%) |
| C03CA04 | Torasemid | 4 (1.9%) |
| L01CD02 | Docetaxel | 4 (1.9%) |
| B01AF01 | Rivaroxaban | 3 (1.4%) |
| L01FA01 | Rituximab | 3 (1.4%) |
| L01FF01 | Nivolumab | 3 (1.4%) |
| N02AX02 | Tramadol | 3 (1.4%) |
| N03AE01 | Clonazepam | 3 (1.4%) |
| N05CF02 | Zolpidem | 3 (1.4%) |
| B01AA04 | Phenprocoumon | 2 (0.9%) |
| J01EE01 | Sulfamethoxazol und trimethoprim | 2 (0.9%) |
| L01BC05 | Gemcitabine | 2 (0.9%) |
| L01BC06 | Capecitabine | 2 (0.9%) |
| L01DB01 | Doxorubicin | 2 (0.9%) |
| L01FF03 | Durvalumab | 2 (0.9%) |
| L01FX25 | Mosunetuzumab | 2 (0.9%) |
| L01XA03 | Oxaliplatin | 2 (0.9%) |
| L01XX52 | Venetoclax | 2 (0.9%) |
| M01AB05 | Diclofenac | 2 (0.9%) |
| M03BX02 | Tizanidine | 2 (0.9%) |
| N02AB03 | Fentanyl | 2 (0.9%) |
| N04BC05 | Pramipexole | 2 (0.9%) |
| N05AD01 | Haloperidol | 2 (0.9%) |
| N05CD08 | Midazolam | 2 (0.9%) |
| N06AB06 | Sertraline | 2 (0.9%) |
| N06AX11 | Mirtazapine | 2 (0.9%) |
| L01AX03 | Temozolomide | 2 (0.9%) |
| N04BA02 | Levodopa and decarboxylase inhibitor | 2 (0.9%) |
| N03AX09 | Lamotrigine | 2 (0.9%) |
| N07BC02 | Methadone | 2 (0.9%) |
| A02BB01 | Misoprostol | 1 (0.5%) |
| A03FA03 | Domperidone | 1 (0.5%) |
| A10AB05 | Insulin aspart | 1 (0.5%) |
| A10AE04 | Insulin glargine | 1 (0.5%) |
| A10AE06 | Insulin degludec | 1 (0.5%) |
| A10BA02 | Metformin | 1 (0.5%) |
| A10BB09 | Gliclazide | 1 (0.5%) |
| B01AB04 | Dalteparin | 1 (0.5%) |
| B01AC06 | Acetylsalicylic acid | 1 (0.5%) |
| B01AC11 | Iloprost | 1 (0.5%) |
| B01AF03 | Edoxaban | 1 (0.5%) |
| B05BC02 | Urea | 1 (0.5%) |
| C01AA05 | Digoxin | 1 (0.5%) |
| C01BD01 | Amiodarone | 1 (0.5%) |
| C03AA03 | Hydrochlorothiazide | 1 (0.5%) |
| C03BA08 | Metolazone | 1 (0.5%) |
| C03DA01 | Spironolactone | 1 (0.5%) |
| C07AB02 | Metoprolol | 1 (0.5%) |
| C07AB07 | Bisoprolol | 1 (0.5%) |
| C07AB12 | Nebivolol | 1 (0.5%) |
| C08CA05 | Nifedipine | 1 (0.5%) |
| C09DX04 | Valsartan and sacubitril | 1 (0.5%) |
| D01AC01 | Clotrimazole | 1 (0.5%) |
| G02BB01 | Vaginal ring with progestogen and estrogen | 1 (0.5%) |
| H02AB07 | Prednisone | 1 (0.5%) |
| H02AB09 | Hydrocortisone | 1 (0.5%) |
| H03AA01 | Levothyroxine sodium | 1 (0.5%) |
| J01AA02 | Doxycycline | 1 (0.5%) |
| J01CR05 | Piperacillin and beta-lactamase-inhibitors | 1 (0.5%) |
| J01XA01 | Vancomycin | 1 (0.5%) |
| J04AB02 | Rifampicin | 1 (0.5%) |
| J05AB15 | Brivudine | 1 (0.5%) |
| L01AA01 | Cyclophosphamide | 1 (0.5%) |
| L01BC07 | Azacitidine | 1 (0.5%) |
| L01CA02 | Vincristine | 1 (0.5%) |
| L01CE01 | Topotecan | 1 (0.5%) |
| L01EF03 | Abemaciclib | 1 (0.5%) |
| L01FF04 | Avelumab | 1 (0.5%) |
| L01FX17 | Sacituzumab govitecan | 1 (0.5%) |
| L01FY02 | Nivolumab and relatlimab | 1 (0.5%) |
| L01XA01 | Cisplatin | 1 (0.5%) |
| L01XX05 | Hydroxycarbamide | 1 (0.5%) |
| L03AB15 | Ropeginterferon alfa-2b | 1 (0.5%) |
| L04AF03 | Upadacitinib | 1 (0.5%) |
| L04AK01 | Leflunomide | 1 (0.5%) |
| L04AX03 | Methotrexate | 1 (0.5%) |
| M01AB15 | Ketorolac | 1 (0.5%) |
| M01AH01 | Celecoxib | 1 (0.5%) |
| M04AC01 | Colchicine | 1 (0.5%) |
| N02AA55 | Oxycodone and naloxone | 1 (0.5%) |
| N02AX06 | Tapentadol | 1 (0.5%) |
| N02BA01 | Acetylsalicylic acid | 1 (0.5%) |
| N03AG01 | Valproic acid | 1 (0.5%) |
| N03AX14 | Levetiracetam | 1 (0.5%) |
| N03AX23 | Brivaracetam | 1 (0.5%) |
| N04AA02 | Biperiden | 1 (0.5%) |
| N05AA02 | Levomepromazine | 1 (0.5%) |
| N05AF03 | Chlorprothixene | 1 (0.5%) |
| N05AH02 | Clozapine | 1 (0.5%) |
| N05AH03 | Olanzapine | 1 (0.5%) |
| N05AX08 | Risperidone | 1 (0.5%) |
| N05AX13 | Paliperidone | 1 (0.5%) |
| N05BA01 | Diazepam | 1 (0.5%) |
| N06AA09 | Amitriptyline | 1 (0.5%) |
| N06AB10 | Escitalopram | 1 (0.5%) |
| N06AX05 | Trazodone | 1 (0.5%) |
| N06AX16 | Venlafaxine | 1 (0.5%) |
| N06DA02 | Donepezil | 1 (0.5%) |
| P01AB01 | Metronidazole | 1 (0.5%) |
| S01CA01 | Dexamethasone and antiinfectives | 1 (0.5%) |
| V08AB05 | Iopromid | 1 (0.5%) |
| L01F | Monoclonal antibodies and antibody drug conjugates | 1 (0.5%) |
| N01AB03 | Methoxyflurane | 1 (0.5%) |
| P02CE01^1^ | Levamisol | 1 (0.5%) |

*^1^ only used in veterinary medicine*

Supplementary Table 3: Characteristics of admissions, stratified by sex with focus on comorbidities.

|  | **Female** | **Male** | **Overall** |
| --- | --- | --- | --- |
| Sex | 113 (56.5) | 87 (43.5) | 200 |
| Age groups (%) |  |  |  |
| 18-49 | 39 (34.5) | 23 (26.4) | 62 (31.0) |
| 50-64 | 28 (24.8) | 24 (27.6) | 52 (26.0) |
| 65-79 | 28 (24.8) | 27 (31.0) | 55 (27.5) |
| ≥ 80 | 18 (15.9) | 13 (14.9) | 31 (15.5) |
| Number of comorbidities (median [IQR]) | 2 [0, 3] | 2 [1, 4] | 2 [1, 3] |
| Congestive heart failure (%) | 7 (6.2) | 8 (9.2) | 15 (7.5) |
| Cardiac arrhythmias (%) | 15 (13.3) | 16 (18.4) | 31 (15.5) |
| Valvular disease (%) | 6 (5.3) | 5 (5.7) | 11 (5.5) |
| Pulmonary circulation_disorders (%) | 1 (0.9) | 2 (2.3) | 3 (1.5) |
| Peripheral vascular disorders (%) | 4 (3.5) | 10 (11.5) | 14 (7.0) |
| Hypertension (uncomplicated) (%) | 15 (13.3) | 17 (19.5) | 32 (16.0) |
| Hypertension (complicated) 1 (%) | 8 (7.1) | 9 (10.3) | 17 (8.5) |
| Paralysis (%) | 2 (1.8) | 3 (3.4) | 5 (2.5) |
| Other neurological disorders (%) | 8 (7.1) | 8 (9.2) | 16 (8.0) |
| Chronic pulmonary disease (%) | 6 (5.3) | 6 (6.9) | 12 (6.0) |
| Diabete (uncomplicated) (%) | 9 (8.0) | 9 (10.3) | 18 (9.0) |
| Diabetes (complicated) (%) | 2 (1.8) | 6 (6.9) | 8 (4.0) |
| Hypothyroidism (%) | 4 (3.5) | 3 (3.4) | 7 (3.5) |
| Renal_failure (%) | 12 (10.6) | 19 (21.8) | 31 (15.5) |
| Liver_disease (%) | 2 (1.8) | 5 (5.7) | 7 (3.5) |
| Peptic ulce disease excluding bleeding_ (%) | 1 (0.9) | 0 (0.0) | 1 (0.5) |
| Lymphoma (%) | 1 (0.9) | 3 (3.4) | 4 (2.0) |
| Metastatic cancer (%) | 14 (12.4) | 8 (9.2) | 22 (11.0) |
| Solid tumor without metastasis (%) | 16 (14.2) | 11 (12.6) | 27 (13.5) |
| Rheumatoid arthritis or collagen vascular disease (%) | 4 (3.5) | 3 (3.4) | 7 (3.5) |
| Coagulopathy (%) | 6 (5.3) | 16 (18.4) | 22 (11.0) |
| Obesity (%) | 2 (1.8) | 6 (6.9) | 8 (4.0) |
| Weight loss (%) | 15 (13.3) | 12 (13.8) | 27 (13.5) |
| Fluid and electrolyte disorders (%) | 23 (20.4) | 26 (29.9) | 49 (24.5) |
| Blood loss anemia (%) | 0 (0.0) | 2 (2.3) | 2 (1.0) |
| Deficiency anemia (%) | 3 (2.7) | 0 (0.0) | 3 (1.5) |
| Alcohol abuse (%) | 7 (6.2) | 7 (8.0) | 14 (7.0) |
| Drug abuse (%) | 6 (5.3) | 11 (12.6) | 17 (8.5) |
| Psychoses (%) | 2 (1.8) | 3 (3.4) | 5 (2.5) |
| Depression (%) | 16 (14.2) | 5 (5.7) | 21 (10.5) |
| Dementia (%) | 3 (2.7) | 3 (3.4) | 6 (3.0) |
| No comorbidities (%) | 35 (31.0) | 8 (9.2) | 43 (21.5) |
| Length of stay in days (median [IQR]) | 3 [1, 7] | 4 [2, 7.50] | 3.50 [1, 7.25] |
| Length of stay in the intensive care unit in hours (median [IQR]) | 0 [0, 5] | 0 [0, 10.50] | 0 [0, 8.25] |
| Length of stay in the intensive care unit in hours (mean [SD]) | 15.22 (63.73) | 21.74(61.17) | 18.06 (62.56) |

Supplementary Table 4: Characteristics of admissions, stratified by sex with focus on drug classes.

|  | **Female** | **Male** | **Overall** |
| --- | --- | --- | --- |
| Sex | 113 (56.5) | 87 (43.5) | 200 |
| Age groups (%) |  |  |  |
| 18-49 | 39 (34.5) | 23 (26.4) | 62 (31.0) |
| 50-64 | 28 (24.8) | 24 (27.6) | 52 (26.0) |
| 65-79 | 28 (24.8) | 27 (31.0) | 55 (27.5) |
| ≥ 80 | 18 (15.9) | 13 (14.9) | 31 (15.5) |
| drug classes (%) |  |  |  |
| Agents acting on the renin-angiotensin system | 1 (0.9) | 0 (0.0) | 1 (0.5) |
| Analgesics without opioids | 8 (7.1) | 7 (8.0) | 15 (7.5) |
| Anesthetics | 0 (0.0) | 1 (1.1) | 1 (0.5) |
| Anti-Parkinson Drugs | 1 (0.9) | 3 (3.4) | 4 (2.0) |
| Antiepileptics | 6 (5.3) | 1 (1.1) | 7 (3.5) |
| Antifungals for dermatological use | 1 (0.9) | 0 (0.0) | 1 (0.5) |
| Antigout preparations | 0 (0.0) | 1 (1.1) | 1 (0.5) |
| Antiinfectives for systemic use | 9 (8.0) | 3 (3.4) | 12 (6.0) |
| Antiinflammatory and antirheumatic products | 4 (3.5) | 2 (2.3) | 6 (3.0) |
| Antimycobacterials | 0 (0.0) | 1 (1.1) | 1 (0.5) |
| Antineoplastic agents | 19 (16.8) | 19 (21.8) | 38 (19.0) |
| Antiprotozoals | 1 (0.9) | 0 (0.0) | 1 (0.5) |
| Antithrombotic agents | 2 (1.8) | 11 (12.6) | 13 (6.5) |
| Antivirals for systemic use | 1 (0.9) | 0 (0.0) | 1 (0.5) |
| Beta blocking agents | 2 (1.8) | 1 (1.1) | 3 (1.5) |
| Blood substitutes and perfusion solutions | 1 (0.9) | 0 (0.0) | 1 (0.5) |
| Calcium channel blockers | 1 (0.9) | 0 (0.0) | 1 (0.5) |
| Cardiac therapy | 2 (1.8) | 0 (0.0) | 2 (1.0) |
| Contrast media | 0 (0.0) | 1 (1.1) | 1 (0.5) |
| Corticosteroids for systemic use | 1 (0.9) | 1 (1.1) | 2 (1.0) |
| Diuretics | 3 (2.7) | 4 (4.6) | 7 (3.5) |
| Drugs for acid related disorders | 1 (0.9) | 0 (0.0) | 1 (0.5) |
| Drugs for functional gastrointestinal disorders | 1 (0.9) | 0 (0.0) | 1 (0.5) |
| Drugs used in Diabetes | 2 (1.8) | 3 (3.4) | 5 (2.5) |
| Immunostimulants | 0 (0.0) | 1 (1.1) | 1 (0.5) |
| Immunosuppressants | 1 (0.9) | 2 (2.3) | 3 (1.5) |
| Muscle relaxants | 0 (0.0) | 2 (2.3) | 2 (1.0) |
| Ophthalmologicals | 1 (0.9) | 0 (0.0) | 1 (0.5) |
| Opioids | 9 (8.0) | 8 (9.2) | 17 (8.5) |
| Other gynecologicals | 1 (0.9) | 0 (0.0) | 1 (0.5) |
| Other nervous system drugs | 1 (0.9) | 0 (0.0) | 1 (0.5) |
| Psychoanaleptics | 4 (3.5) | 1 (1.1) | 5 (2.5) |
| Psycholeptics | 20 (17.7) | 8 (9.2) | 28 (14.0) |
| Thyroid therapy | 1 (0.9) | 0 (0.0) | 1 (0.5) |
| More than one drug | 7 (6.2) | 4 (4.6) | 11 (5.5) |
| Other drugs | 1 (0.9) | 2 (2.3) | 3 (1.5) |
| Concomitant drugs (%) |  |  |  |
| no concomitant drug suspected (%) | 72 (63.7) | 66 (75.9) | 138 (69.0) |
| other concomitant drug suspected | 38 (33.6) | 20 (23.0) | 58 (29.0) |
| opioid as concomitant drug suspected | 3 (2.7) | 1 (1.1) | 4 (2.0) |
| Length of stay in days (median [IQR]) | 3 [1, 7] | 4 [2, 7.50] | 3.50 [1, 7.25] |
| Length of stay in the intensive care unit in hours (median [IQR]) | 0 [0, 5] | 0 [0, 10.50] | 0 [0, 8.25] |
| Length of stay in the intensive care unit in hours (mean [SD]) (mean [SD]) | 15.22(63.73) | 21.74(61.17) | 18.06 (62.56) |

Supplementary Table 5: Characteristics of admissions, stratified by sex with focus on ICD-10 chapters of diagnoses for ADRs.

|  | **Female** | **Male** | **Overall** |
| --- | --- | --- | --- |
| N | 113 (56.5) | 87 (43.5) | 200 |
| Age groups (%) |  |  |  |
| 18-49 | 39 (34.5) | 23 (26.4) | 62 (31.0) |
| 50-64 | 28 (24.8) | 24 (27.6) | 52 (26.0) |
| 65-79 | 28 (24.8) | 27 (31.0) | 55 (27.5) |
| ≥ 80 | 18 (15.9) | 13 (14.9) | 31 (15.5) |
| ICD10chapter diagnosis (%) |  |  |  |
| A00-B99: Certain infectious and parasitic diseases | 2 (1.8) | 1 (1.1) | 3 (1.5) |
| D50-D89: Diseases of the blood and blood-forming organs and certain disorders involving the immune mechanism | 9 (8.0) | 12 (13.8) | 21 (10.5) |
| E00-E90: Endocrine, nutritional and metabolic diseases | 4 (3.5) | 6 (6.9) | 10 (5.0) |
| F01-F99: Mental and behavioural disorders | 6 (5.3) | 8 (9.2) | 14 (7.0) |
| G00-G99: Diseases of the nervous system | 0 (0.0) | 6 (6.9) | 6 (3.0) |
| H00-H59: Diseases of the eye and adnexa | 1 (0.9) | 0 (0.0) | 1 (0.5) |
| I00-I99: Diseases of the circulatory system | 5 (4.4) | 3 (3.4) | 8 (4.0) |
| J00-J99: Diseases of the respiratory system | 4 (3.5) | 4 (4.6) | 8 (4.0) |
| K00-K93: Diseases of the digestive system | 18 (15.9) | 11 (12.6) | 29 (14.5) |
| L00-L99: Diseases of the skin and subcutaneous tissue | 7 (6.2) | 1 (1.1) | 8 (4.0) |
| M00-M99: Diseases of the musculoskeletal system and connective tissue | 0 (0.0) | 1 (1.1) | 1 (0.5) |
| N00-N99: Diseases of the genitourinary system | 0 (0.0) | 2 (2.3) | 2 (1.0) |
| R00-R99: Symptoms, signs and abnormal clinical and laboratory findings, not elsewhere classified | 11 (9.7) | 12 (13.8) | 23 (11.5) |
| S00-T98: Injury, poisoning and certain other consequences of external causes | 44 (38.9) | 20 (23.0) | 64 (32.0) |
| U00-U85: Codes for special purposes | 1 (0.9) | 0 (0.0) | 1 (0.5) |
| Z00-Z99: Factors influencing health status and contact with health services | 1 (0.9) | 0 (0.0) | 1 (0.5) |
| Length of stay in days (median [IQR]) | 3 [1, 7] | 4 [2, 7.50] | 3.50 [1, 7.25] |
| Length of stay in the intensive care unit in hours (median [IQR]) | 0 [0, 5] | 0 [0, 10.50] | 0 [0, 8.25] |
| Length of stay in the intensive care unit in hours (mean[SD]) | 15.22(63.73) | 21.74(61.17) | 0.467 |

Supplementary Table 6: Characteristics of admissions, stratified by ICD-10 code chapters for ADRs.

|  | **A00-B99** | **D50-D89** | **E00-E90** | **F01-F99** | **G00-G99** | **H00-H59** | **I00-I99** | **J00-J99** | **K00-K93** | **L00-L99** | **M00-M99** | **N00-N99** | **R00-R99** | **S00-T98** | **U00-U85** | **Z00-Z99** |
| --- | --- | --- | --- | --- | --- | --- | --- | --- | --- | --- | --- | --- | --- | --- | --- | --- |
| **N ICD-10 coded ADR** | 3 (1.5) | 21 (10.5) | 10 (5) | 14 (7) | 6 (3) | 1 (0.5) | 8 (4) | 8 (4) | 29 (14.5) | 8 (4) | 1 (0.5) | 2 (1) | 23 (11.5) | 64 (32) | 1 (0.5) | 1 (0.5) |
| **Age groups (%)** |  |  |  |  |  |  |  |  |  |  |  |  |  |  |  |  |
| 18-49 | 0 (0.0) | 8 (38.1) | 1 (10.0) | 12 (85.7) | 1 (16.7) | 0 (0.0) | 2 (25.0) | 0 (0.0) | 1 (3.4) | 2 (25.0) | 0 (0.0) | 0 (0.0) | 4 (17.4) | 31 (48.4) | 0 (0.0) | 0 (0.0) |
| 50-64 | 0 (0.0) | 0 (0.0) | 2 (20.0) | 1 (7.1) | 2 (33.3) | 0 (0.0) | 0 (0.0) | 4 (50.0) | 13 (44.8) | 4 (50.0) | 0 (0.0) | 1 (50.0) | 7 (30.4) | 17 (26.6) | 0 (0.0) | 1 (100.0) |
| 65-79 | 2 (66.7) | 7 (33.3) | 5 (50.0) | 1 (7.1) | 3 (50.0) | 1 (100.0) | 4 (50.0) | 4 (50.0) | 6 (20.7) | 2 (25.0) | 1 (100.0) | 1 (50.0) | 9 (39.1) | 9 (14.1) | 0 (0.0) | 0 (0.0) |
| ≥ 80 | 1 (33.3) | 6 (28.6) | 2 (20.0) | 0 (0.0) | 0 (0.0) | 0 (0.0) | 2 (25.0) | 0 (0.0) | 9 (31.0) | 0 (0.0) | 0 (0.0) | 0 (0.0) | 3 (13.0) | 7 (10.9) | 1 (100.0) | 0 (0.0) |
| **Sex, male (%)** | 1 (33.3) | 12 (57.1) | 6 (60.0) | 8 (57.1) | 6 (100.0) | 0 (0.0) | 3 (37.5) | 4 (50.0) | 11 (37.9) | 1 (12.5) | 1 (100.0) | 2 (100.0) | 12 (52.2) | 20 (31.2) | 0 (0.0) | 0 (0.0) |
| **Drug classes (%)** |  |  |  |  |  |  |  |  |  |  |  |  |  |  |  |  |
| Agents acting on the renin-angiotensin system | 0 (0.0) | 0 (0.0) | 0 (0.0) | 0 (0.0) | 0 (0.0) | 0 (0.0) | 0 (0.0) | 0 (0.0) | 1 (3.4) | 0 (0.0) | 0 (0.0) | 0 (0.0) | 0 (0.0) | 0 (0.0) | 0 (0.0) | 0 (0.0) |
| Analgesics without opioids | 0 (0.0) | 3 (14.3) | 0 (0.0) | 0 (0.0) | 1 (16.7) | 0 (0.0) | 1 (12.5) | 0 (0.0) | 2 (6.9) | 0 (0.0) | 0 (0.0) | 0 (0.0) | 0 (0.0) | 8 (12.5) | 0 (0.0) | 0 (0.0) |
| Anesthetics | 0 (0.0) | 0 (0.0) | 0 (0.0) | 0 (0.0) | 0 (0.0) | 0 (0.0) | 0 (0.0) | 0 (0.0) | 0 (0.0) | 0 (0.0) | 0 (0.0) | 0 (0.0) | 0 (0.0) | 1 (1.6) | 0 (0.0) | 0 (0.0) |
| Anti-Parkinson Drugs | 0 (0.0) | 0 (0.0) | 0 (0.0) | 0 (0.0) | 2 (33.3) | 0 (0.0) | 0 (0.0) | 0 (0.0) | 0 (0.0) | 0 (0.0) | 0 (0.0) | 0 (0.0) | 2 (8.7) | 0 (0.0) | 0 (0.0) | 0 (0.0) |
| Antiepileptics | 0 (0.0) | 0 (0.0) | 0 (0.0) | 1 (7.1) | 0 (0.0) | 0 (0.0) | 0 (0.0) | 0 (0.0) | 0 (0.0) | 0 (0.0) | 0 (0.0) | 0 (0.0) | 2 (8.7) | 4 (6.2) | 0 (0.0) | 0 (0.0) |
| Antifungals for dermatological use | 0 (0.0) | 0 (0.0) | 0 (0.0) | 0 (0.0) | 0 (0.0) | 0 (0.0) | 0 (0.0) | 0 (0.0) | 0 (0.0) | 1 (12.5) | 0 (0.0) | 0 (0.0) | 0 (0.0) | 0 (0.0) | 0 (0.0) | 0 (0.0) |
| Antigout preparations | 0 (0.0) | 0 (0.0) | 1 (10.0) | 0 (0.0) | 0 (0.0) | 0 (0.0) | 0 (0.0) | 0 (0.0) | 0 (0.0) | 0 (0.0) | 0 (0.0) | 0 (0.0) | 0 (0.0) | 0 (0.0) | 0 (0.0) | 0 (0.0) |
| Antiinfectives for systemic use | 3 (100.0) | 0 (0.0) | 0 (0.0) | 0 (0.0) | 0 (0.0) | 0 (0.0) | 0 (0.0) | 0 (0.0) | 3 (10.3) | 2 (25.0) | 0 (0.0) | 0 (0.0) | 2 (8.7) | 2 (3.1) | 0 (0.0) | 0 (0.0) |
| Antiinflammatory and antirheumatic products | 0 (0.0) | 0 (0.0) | 0 (0.0) | 0 (0.0) | 0 (0.0) | 0 (0.0) | 0 (0.0) | 1 (12.5) | 3 (10.3) | 0 (0.0) | 0 (0.0) | 0 (0.0) | 0 (0.0) | 2 (3.1) | 0 (0.0) | 0 (0.0) |
| Antimycobacterials | 0 (0.0) | 0 (0.0) | 0 (0.0) | 0 (0.0) | 0 (0.0) | 0 (0.0) | 0 (0.0) | 0 (0.0) | 0 (0.0) | 0 (0.0) | 1 (100.0) | 0 (0.0) | 0 (0.0) | 0 (0.0) | 0 (0.0) | 0 (0.0) |
| Antineoplastic agents | 0 (0.0) | 8 (38.1) | 3 (30.0) | 1 (7.1) | 0 (0.0) | 0 (0.0) | 0 (0.0) | 6 (75.0) | 12 (41.4) | 2 (25.0) | 0 (0.0) | 0 (0.0) | 5 (21.7) | 1 (1.6) | 0 (0.0) | 0 (0.0) |
| Antiprotozoals | 0 (0.0) | 0 (0.0) | 0 (0.0) | 0 (0.0) | 0 (0.0) | 0 (0.0) | 0 (0.0) | 0 (0.0) | 0 (0.0) | 0 (0.0) | 0 (0.0) | 0 (0.0) | 1 (4.3) | 0 (0.0) | 0 (0.0) | 0 (0.0) |
| Antithrombotic agents | 0 (0.0) | 7 (33.3) | 0 (0.0) | 0 (0.0) | 0 (0.0) | 0 (0.0) | 0 (0.0) | 0 (0.0) | 3 (10.3) | 0 (0.0) | 0 (0.0) | 0 (0.0) | 1 (4.3) | 2 (3.1) | 0 (0.0) | 0 (0.0) |
| Antivirals for systemic use | 0 (0.0) | 0 (0.0) | 0 (0.0) | 0 (0.0) | 0 (0.0) | 0 (0.0) | 0 (0.0) | 0 (0.0) | 1 (3.4) | 0 (0.0) | 0 (0.0) | 0 (0.0) | 0 (0.0) | 0 (0.0) | 0 (0.0) | 0 (0.0) |
| Beta blocking agents | 0 (0.0) | 0 (0.0) | 0 (0.0) | 0 (0.0) | 0 (0.0) | 0 (0.0) | 2 (25.0) | 0 (0.0) | 0 (0.0) | 0 (0.0) | 0 (0.0) | 0 (0.0) | 0 (0.0) | 1 (1.6) | 0 (0.0) | 0 (0.0) |
| Blood substitutes and perfusion solutions | 0 (0.0) | 0 (0.0) | 0 (0.0) | 0 (0.0) | 0 (0.0) | 0 (0.0) | 0 (0.0) | 0 (0.0) | 0 (0.0) | 0 (0.0) | 0 (0.0) | 0 (0.0) | 1 (4.3) | 0 (0.0) | 0 (0.0) | 0 (0.0) |
| Calcium channel blockers | 0 (0.0) | 0 (0.0) | 0 (0.0) | 0 (0.0) | 0 (0.0) | 0 (0.0) | 0 (0.0) | 0 (0.0) | 0 (0.0) | 0 (0.0) | 0 (0.0) | 0 (0.0) | 0 (0.0) | 1 (1.6) | 0 (0.0) | 0 (0.0) |
| Cardiac therapy | 0 (0.0) | 0 (0.0) | 0 (0.0) | 0 (0.0) | 0 (0.0) | 0 (0.0) | 1 (12.5) | 0 (0.0) | 0 (0.0) | 0 (0.0) | 0 (0.0) | 0 (0.0) | 0 (0.0) | 1 (1.6) | 0 (0.0) | 0 (0.0) |
| Contrast media | 0 (0.0) | 0 (0.0) | 0 (0.0) | 0 (0.0) | 0 (0.0) | 0 (0.0) | 0 (0.0) | 0 (0.0) | 0 (0.0) | 0 (0.0) | 0 (0.0) | 0 (0.0) | 1 (4.3) | 0 (0.0) | 0 (0.0) | 0 (0.0) |
| Corticosteroids for systemic use | 0 (0.0) | 0 (0.0) | 1 (10.0) | 0 (0.0) | 0 (0.0) | 0 (0.0) | 0 (0.0) | 0 (0.0) | 0 (0.0) | 0 (0.0) | 0 (0.0) | 0 (0.0) | 0 (0.0) | 1 (1.6) | 0 (0.0) | 0 (0.0) |
| Diuretics | 0 (0.0) | 0 (0.0) | 4 (40.0) | 0 (0.0) | 0 (0.0) | 0 (0.0) | 1 (12.5) | 0 (0.0) | 0 (0.0) | 0 (0.0) | 0 (0.0) | 2 (100.0) | 0 (0.0) | 0 (0.0) | 0 (0.0) | 0 (0.0) |
| Drugs for acid related disorders | 0 (0.0) | 1 (4.8) | 0 (0.0) | 0 (0.0) | 0 (0.0) | 0 (0.0) | 0 (0.0) | 0 (0.0) | 0 (0.0) | 0 (0.0) | 0 (0.0) | 0 (0.0) | 0 (0.0) | 0 (0.0) | 0 (0.0) | 0 (0.0) |
| Drugs for functional gastrointestinal disorders | 0 (0.0) | 0 (0.0) | 0 (0.0) | 0 (0.0) | 0 (0.0) | 0 (0.0) | 0 (0.0) | 0 (0.0) | 0 (0.0) | 0 (0.0) | 0 (0.0) | 0 (0.0) | 0 (0.0) | 1 (1.6) | 0 (0.0) | 0 (0.0) |
| Drugs used in Diabetes | 0 (0.0) | 0 (0.0) | 1 (10.0) | 0 (0.0) | 0 (0.0) | 0 (0.0) | 0 (0.0) | 0 (0.0) | 0 (0.0) | 0 (0.0) | 0 (0.0) | 0 (0.0) | 0 (0.0) | 4 (6.2) | 0 (0.0) | 0 (0.0) |
| Immunostimulants | 0 (0.0) | 0 (0.0) | 0 (0.0) | 0 (0.0) | 0 (0.0) | 0 (0.0) | 0 (0.0) | 0 (0.0) | 0 (0.0) | 0 (0.0) | 0 (0.0) | 0 (0.0) | 1 (4.3) | 0 (0.0) | 0 (0.0) | 0 (0.0) |
| Immunosuppressants | 0 (0.0) | 1 (4.8) | 0 (0.0) | 0 (0.0) | 0 (0.0) | 0 (0.0) | 0 (0.0) | 1 (12.5) | 1 (3.4) | 0 (0.0) | 0 (0.0) | 0 (0.0) | 0 (0.0) | 0 (0.0) | 0 (0.0) | 0 (0.0) |
| Muscle relaxants | 0 (0.0) | 0 (0.0) | 0 (0.0) | 0 (0.0) | 0 (0.0) | 0 (0.0) | 1 (12.5) | 0 (0.0) | 0 (0.0) | 0 (0.0) | 0 (0.0) | 0 (0.0) | 0 (0.0) | 1 (1.6) | 0 (0.0) | 0 (0.0) |
| Ophthalmologicals | 0 (0.0) | 0 (0.0) | 0 (0.0) | 0 (0.0) | 0 (0.0) | 1 (100.0) | 0 (0.0) | 0 (0.0) | 0 (0.0) | 0 (0.0) | 0 (0.0) | 0 (0.0) | 0 (0.0) | 0 (0.0) | 0 (0.0) | 0 (0.0) |
| Opioids | 0 (0.0) | 0 (0.0) | 0 (0.0) | 3 (21.4) | 1 (16.7) | 0 (0.0) | 0 (0.0) | 0 (0.0) | 1 (3.4) | 1 (12.5) | 0 (0.0) | 0 (0.0) | 5 (21.7) | 5 (7.8) | 0 (0.0) | 1 (100.0) |
| Other gynecologicals | 0 (0.0) | 0 (0.0) | 0 (0.0) | 0 (0.0) | 0 (0.0) | 0 (0.0) | 1 (12.5) | 0 (0.0) | 0 (0.0) | 0 (0.0) | 0 (0.0) | 0 (0.0) | 0 (0.0) | 0 (0.0) | 0 (0.0) | 0 (0.0) |
| Other nervous system drugs | 0 (0.0) | 0 (0.0) | 0 (0.0) | 0 (0.0) | 0 (0.0) | 0 (0.0) | 0 (0.0) | 0 (0.0) | 0 (0.0) | 0 (0.0) | 0 (0.0) | 0 (0.0) | 0 (0.0) | 1 (1.6) | 0 (0.0) | 0 (0.0) |
| Psychoanaleptics | 0 (0.0) | 0 (0.0) | 0 (0.0) | 1 (7.1) | 0 (0.0) | 0 (0.0) | 0 (0.0) | 0 (0.0) | 0 (0.0) | 0 (0.0) | 0 (0.0) | 0 (0.0) | 1 (4.3) | 3 (4.7) | 0 (0.0) | 0 (0.0) |
| Psycholeptics | 0 (0.0) | 0 (0.0) | 0 (0.0) | 6 (42.9) | 2 (33.3) | 0 (0.0) | 0 (0.0) | 0 (0.0) | 0 (0.0) | 0 (0.0) | 0 (0.0) | 0 (0.0) | 0 (0.0) | 19 (29.7) | 1 (100.0) | 0 (0.0) |
| Thyroid therapy | 0 (0.0) | 0 (0.0) | 0 (0.0) | 0 (0.0) | 0 (0.0) | 0 (0.0) | 1 (12.5) | 0 (0.0) | 0 (0.0) | 0 (0.0) | 0 (0.0) | 0 (0.0) | 0 (0.0) | 0 (0.0) | 0 (0.0) | 0 (0.0) |
| more than one drug | 0 (0.0) | 1 (4.8) | 0 (0.0) | 2 (14.3) | 0 (0.0) | 0 (0.0) | 0 (0.0) | 0 (0.0) | 2 (6.9) | 1 (12.5) | 0 (0.0) | 0 (0.0) | 0 (0.0) | 5 (7.8) | 0 (0.0) | 0 (0.0) |
| other drugs | 0 (0.0) | 0 (0.0) | 0 (0.0) | 0 (0.0) | 0 (0.0) | 0 (0.0) | 0 (0.0) | 0 (0.0) | 0 (0.0) | 1 (12.5) | 0 (0.0) | 0 (0.0) | 1 (4.3) | 1 (1.6) | 0 (0.0) | 0 (0.0) |
| **Concomitant drugs** |  |  |  |  |  |  |  |  |  |  |  |  |  |  |  |  |
| no concomitant drug suspected (%) | 2 (66.7) | 16 (76.2) | 10 (100.0) | 6 (42.9) | 2 (33.3) | 1 (100.0) | 8 (100.0) | 8 (100.0) | 17 (58.6) | 6 (75.0) | 1 (100.0) | 1 (50.0) | 17 (73.9) | 43 (67.2) | 0 (0.0) | 0 (0.0) |
| other concomitant drug suspected (%) | 1 (33.3) | 5 (23.8) | 0 (0.0) | 8 (57.1) | 3 (50.0) | 0 (0.0) | 0 (0.0) | 0 (0.0) | 12 (41.4) | 2 (25.0) | 0 (0.0) | 1 (50.0) | 6 (26.1) | 18 (28.1) | 1 (100.0) | 1 (100.0) |
| opioid as concomitant drug suspected (%) | 0 (0.0) | 0 (0.0) | 0 (0.0) | 0 (0.0) | 1 (16.7) | 0 (0.0) | 0 (0.0) | 0 (0.0) | 0 (0.0) | 0 (0.0) | 0 (0.0) | 0 (0.0) | 0 (0.0) | 3 (4.7) | 0 (0.0) | 0 (0.0) |
| **Length of stay in days (median [IQR])** | 4 [4, 4.50] | 7 [4, 13] | 7 [5, 14] | 1 [1, 2] | 7.50 [3.50, 13] | 2 [2, 2] | 3.50 [2, 5.25] | 14 [12, 16] | 6 [3, 7] | 4.50 [2.50, 8.75] | 4 [4, 4] | 9 [8, 10] | 5 [2.50, 14] | 1 [1, 3] | 4 [4, 4] | 1 [1, 1] |
| **Length of stay in the intensive care unit in hours (median [IQR])** | 0 [0, 0] | 0 [0, 12] | 0 [0, 50.75] | 14 [0, 16] | 0 [0, 0] | 0 [0, 0] | 0 [0, 12.25] | 26.50 [0, 203.25] | 0 [0, 0] | 0 [0, 0] | 0 [0, 0] | 0 [0, 0] | 0 [0, 0] | 0 [0, 15] | 0 [0, 0] | 0 [0, 0] |
| **Length of stay in the intensive care unit in hours (mean[SD])** | 0 (0) | 26.29 (57.65) | 29.40 (44.24) | 14.43 (17.07) | 2.5(6.12) | 0 (NA) | 71.25 (190.72) | 110.25 (148.97) | 0.93 (4.02) | 0.62 (1.77) | 0 | 0(0) | 18.09 (79.65) | 10.12 (21.01) | 0 | 0 |

Supplementary Table 7: Overview of all ICD-10 coded ADRs that led to hospitalization, grouped by ICD-10 code chapters.

| **ADR ICD-10 code** | **Description** | **N (% of total [n=200])** |
| --- | --- | --- |
| **S00-T98** | **Injury, poisoning and certain other consequences of external causes** | **64 (32%)** |
| T42.4 | Poisoning by benzodiazepines | 10 |
| T39.1 | Poisoning by 4-Aminophenol derivatives | 8 |
| T43.5 | Poisoning by other and unspecified antipsychotics and neuroleptics | 6 |
| T40.2 | Poisoning by other opioids | 5 |
| T43.2 | Poisoning by other and unspecified antidepressants | 5 |
| T38.3 | Poisoning by insulin and oral hypoglycaemic (antidiabetic) drugs | 4 |
| T42.6 | Poisoning by other antiepileptic and sedative-hypnotic drugs | 3 |
| T39.3 | Poisoning by other nonsteroidal anti-inflammatory drugs (NSAID) | 3 |
| T43.4 | Poisoning by butyrophenone and thioxanthene neuroleptics | 2 |
| T88.6 | Anaphylactic shock due to adverse effect of correct drug or medicament properly administered | 2 |
| T78.3 | Angioneurotic oedema | 2 |
| T88.7 | Unspecified adverse effect of drug or medicament | 2 |
| T39.2 | Poisoning by pyrazolone derivatives | 1 |
| T40.4 | Poisoning by other synthetic narcotics | 1 |
| T42.8 | Poisoning by antiparkinsonism drugs and other central muscle-tone depressants | 1 |
| T44.7 | Poisoning by predominantly alpha-adrenoreceptor agonists, not elsewhere classified | 1 |
| T46.0 | Poisoning by cardiac-stimulant glycosides and drugs of similar action | 1 |
| T46.1 | Poisoning by calcium-channel blockers | 1 |
| T43.3 | Poisoning by phenothiazine antipsychotics and neuroleptics | 1 |
| T80.6 | Other serum reactions | 1 |
| S70.0 | Contusion of hip | 1 |
| T38.2 | Poisoning by antithyroid drugs | 1 |
| T40.3 | Poisoning by methadone | 1 |
| T42.7 | Poisoning by antiepileptic and sedative-hypnotic drugs, unspecified | 1 |
| **K00-K93** | **Diseases of the digestive system** | **29 (14.5%)** |
| K52.1 | Toxic gastroenteritis and colitis | 11 |
| K25.0 | Gastric ulcer acute with haemorrhage | 2 |
| K26.0 | Duodenal ulcer acute with haemorrhage | 2 |
| K52.9 | Noninfective gastroenteritis and colitis, unspecified | 2 |
| K71.0 | Toxic liver disease with cholestasis | 2 |
| K25.4 | Gastric ulcer chronic or unspecified with haemorrhage | 1 |
| K26.4 | Duodenal ulcer chronic or unspecified with haemorrhage | 1 |
| K29.5 | Chronic gastritis, unspecified | 1 |
| K56.4 | Other impaction of intestine | 1 |
| K62.5 | Haemorrhage of anus and rectum | 1 |
| K71.2 | Toxic liver disease with acute hepatitis | 1 |
| K71.8 | Toxic liver disease with other disorders of liver | 1 |
| K75.8 | Other specified inflammatory liver diseases | 1 |
| K83.0 | Cholangitis | 1 |
| K92.1 | Melaena | 1 |
| **R00-R99** | **Symptoms, signs and abnormal clinical and laboratory findings, not elsewhere classified** | **23 (11.5%)** |
| R11 | Nausea and vomiting | 10 |
| R40.0 | Somnolence | 2 |
| R25.1 | Tremor, unspecified | 1 |
| R26.8 | Other and unspecified abnormalities of gait and mobility | 1 |
| R31 | Unspecified haematuria | 1 |
| R33 | Retention of urine | 1 |
| R43.2 | Parageusia | 1 |
| R44.0 | Auditory hallucinations | 1 |
| R50.2 | Drug-induced fever | 1 |
| R55 | Syncope and collapse | 1 |
| R57.2 | Septic shock | 1 |
| R57.9 | Shock, unspecified | 1 |
| R63.0 | Anorexia | 1 |
| **D50-D89** | **Diseases of the blood and blood-forming organs and certain disorders involving the immune mechanism** | **21 (10.5%)** |
| D70.1 | Drug-induced agranulocytosis and neutropenia | 8 |
| D68.3 | Haemorrhagic disorder due to circulating anticoagulants | 6 |
| D76.4 | Cytokine release syndrome | 3 |
| D62 | Acute posthaemorrhagic anaemia | 1 |
| D68.4 | Acquired coagulation factor deficiency | 1 |
| D69.6 | Thrombocytopenia, unspecified | 1 |
| D70.3 | Neutropenia due to infection | 1 |
| **F01-F99** | **Mental and behavioural disorders** | **14 (7%)** |
| F13.0 | Mental and behavioural disorders due to use of sedatives or hypnotics - Acute intoxication | 3 |
| F11.0 | Mental and behavioural disorders due to use of opioids - Acute intoxication | 2 |
| F11.4 | Mental and behavioural disorders due to use of opioids - Withdrawal state with delirium | 2 |
| F13.1 | Mental and behavioural disorders due to use of sedatives or hypnotics - Harmful use | 2 |
| F11.2 | Mental and behavioural disorders due to use of opioids - Dependence syndrome | 1 |
| F11.3 | Mental and behavioural disorders due to use of opioids - Withdrawal state | 1 |
| F15.0 | Mental and behavioural disorders due to use of other stimulants, including caffeine - Acute intoxication | 1 |
| F19.0 | Mental and behavioural disorders due to multiple drug use and use of other psychoactive substances - Acue Intoxication | 1 |
| F99 | Mental disorder, not otherwise specified | 1 |
| **E00-E90** | **Endocrine, nutritional and metabolic diseases** | **10 (5%)** |
| E87.1 | Hypo-osmolality and hyponatraemia | 4 |
| E13.9 | Other specified diabetes mellitus without complications | 1 |
| E22.2 | Syndrome of inappropriate secretion of antidiuretic hormone | 1 |
| E23.6 | Other disorders of pituitary gland | 1 |
| E27.3 | Drug-induced adrenocortical insufficiency | 1 |
| E87.2 | Acidosis | 1 |
| E87.6 | Hypokalaemia | 1 |
| **I00-I99** | **Diseases of the circulatory system** | **8 (4%)** |
| I95.2 | Hypotension due to drugs | 4 |
| I45.2 | Bifascicular block | 1 |
| I48.0 | Paroxysmal atrial fibrillation | 1 |
| I49.8 | Other specified cardiac arrhythmias | 1 |
| I67.6 | Nonpyogenic thrombosis of intracranial venous system | 1 |
| **J00-J99** | **Diseases of the respiratory system** | **8 (4%)** |
| J70.2 | Acute drug-induced interstitial lung disorders | 6 |
| J12.8 | Other viral pneumonia | 1 |
| J45.9 | Asthma, unspecified | 1 |
| **L00-L99** | **Diseases of the skin and subcutaneous tissue** | **8 (4%)** |
| L27.0 | Generalized skin eruption due to drugs and medicaments | 4 |
| L27.1 | Localized skin eruption due to drugs and medicaments | 1 |
| L29.8 | Other pruritus | 1 |
| L50.0 | Allergic urticaria | 1 |
| L51.8 | Other erythema multiforme | 1 |
| **G00-G99** | **Diseases of the nervous system** | **6 (3%)** |
| G24.0 | Drug-induced dystonia | 2 |
| G21.0 | Secondary parkinsonism | 1 |
| G25.8 | Other specified extrapyramidal and movement disorders | 1 |
| G44.4 | Drug-induced headache, not elsewhere classified | 1 |
| G93.6 | Cerebral oedema | 1 |
| **A00-B99** | **Certain infectious and parasitic diseases** | **3 (1.5%)** |
| A04.7 | Enterocolitis due to Clostridium difficile | 3 |
| **N00-N99** | **Diseases of the genitourinary system** | **2 (1%)** |
| N18.3 | Chronic kidney disease, stage 3 | 1 |
| N18.5 | Chronic kidney disease, stage 5 | 1 |
|  | **Remaining diagnoses** | **4 (2%)** |
| H40.6 | Glaucoma secondary to drugs | 1 |
| M54.8 | Other dorsalgia | 1 |
| U51.2 | Severe cognitive impairment | 1 |
| Z03.6 | Observation for suspected toxic effect from ingested substance | 1 |

## Supplementary Methods: ICD-10-Based Logic for Identifying ADR-Related Admissions

This supplementary material details the ICD-10-based identification strategy used to detect ADR-related hospitalizations. The logic builds on previously published approaches used in Swiss administrative data and international ICD-10-based ADR detection frameworks. It integrates three complementary detection pathways to maximize sensitivity: explicit drug-induced diagnoses, supplementary ADR-indicative codes, and medication poisoning codes.

**To acknowledge use of this resource, please cite:**

Weber GA, Bartel S, Boch M, Rosen C, Bodmer M, Hug BL, Stammschulte T, Beeler PE.

Increasing the reporting of adverse drug reaction-related hospitalizations using an ICD-10-based identification workflow: A multicentre study from Switzerland.

*Br J Clin Pharmacol*. 2026. doi: 10.1002/bcp.70564.

**Please also cite the original publication that introduced the ICD‑10-based identification logic:**

Beeler PE, Stammschulte T, Dressel H.

Hospitalisations Related to Adverse Drug Reactions in Switzerland in 2012-2019: Characteristics, In-Hospital Mortality, and Spontaneous Reporting Rate.

*Drug Saf*. 2023 Aug;46(8):753-763. doi: 10.1007/s40264-023-01319-y.

**Explanation:**

Hospitalizations were classified as related to ADRs by applying the Swiss ICD-10-GM coding rules. These rules allow ADRs to be represented either through primary diagnoses that explicitly denote medication-induced conditions or through supplementary ICD-10 codes that link a clinical condition to a medicinal product.

First, admissions were included when the primary diagnosis described a condition known to be caused by a drug or vaccine. This category encompassed all ICD-10 codes that explicitly specify a drug-induced etiology or poisoning by a medicinal product.

Second, admissions were included when the primary diagnosis was accompanied by ADR-indicative supplementary codes. According to Swiss coding rules, the ICD-10 codes Y57.9 and Y59.9 indicate that the clinical condition coded as the primary diagnosis was triggered by a drug or a biological product. In Swiss administrative data, “ancillary information” is a structured extension to the primary diagnosis and is coded by hospital coding specialists. It is distinct from secondary diagnoses. These supplementary codes may appear either in the ancillary information field or, in some cases, as the first secondary diagnosis, and both placements were treated equivalently.

Third, poisoning codes related to medicinal products, primarily those within the T36–T50 range, along with selected additional T codes representing acute drug-related reactions, were classified as ADRs.

By combining these three elements, explicit drug-induced diagnoses, ADR-indicative supplementary codes, and medication-related poisoning codes, the identification strategy captures all hospitalizations in which a drug was the likely cause of the clinical condition leading to admission. A full list of all ICD-10 codes used is presented in the SQL WHERE clause below, which operationalizes this detection logic.

| 1 | -- Comprehensive SQL WHERE clause | | | | | | | | | | |
| --- | --- | --- | --- | --- | --- | --- | --- | --- | --- | --- | --- |
| 2 |  | | | | | | | | | | |
| 3 | -- PD = primary diagnosis | | | | | | | | | | |
| 4 | -- ANC = ancillary information | | | | | | | | | |  |
| 5 | -- SD1 = first secondary diagnosis | | | | | | | | | | |
| 6 |  | |  |  |  | | | | | | |
| 7 | **WHERE** | |  |  |  | | | | | | |
| 8 | **(** | |  |  |  | | | | | | |
| 9 | -- | | Primary diagnosis | | | | | path | (221 | prefixes) | |
| 10 | PD | | **LIKE** | 'A047%' **OR** | | | | | | | |
| 11 | PD | | **LIKE** | 'A800%' **OR** | | | | | | | |
| 12 | PD | | **LIKE** | 'D521%' **OR** | | | | | | | |
| 13 | PD | | **LIKE** | 'D590%' **OR** | | | | | | | |
| 14 | PD | | **LIKE** | 'D592%' **OR** | | | | | | | |
| 15 | PD | | **LIKE** | 'D611%' **OR** | | | | | | | |
| 16 | PD | | **LIKE** | 'D62%' | **OR** | | | | | | |
| 17 | PD | | **LIKE** | 'D642%' **OR** | | | | | | | |
| 18 | PD | | **LIKE** | 'D683%' **OR** | | | | | | | |
| 19 | PD | | **LIKE** | 'D695%' **OR** | | | | | | | |
| 20 | PD | | **LIKE** | 'D696%' **OR** | | | | | | | |
| 21 | PD | | **LIKE** | 'D70%' | **OR** | | | | | | |
| 22 | PD | | **LIKE** | 'E032%' **OR** | | | | | | | |
| 23 | PD | | **LIKE** | 'E064%' **OR** | | | | | | | |
| 24 | PD | | **LIKE** | 'E15%' | **OR** | | | | | | |
| 25 | PD | | **LIKE** | 'E160%' **OR** | | | | | | | |
| 26 | PD | | **LIKE** | 'E231%' **OR** | | | | | | | |
| 27 | PD | | **LIKE** | 'E242%' **OR** | | | | | | | |
| 28 | PD | | **LIKE** | 'E273%' **OR** | | | | | | | |
| 29 | PD | | **LIKE** | 'E661%' **OR** | | | | | | | |
| 30 | PD | | **LIKE** | 'F11%' | **OR** | | | | | | |
| 31 | PD | | **LIKE** | 'F13%' | **OR** | | | | | | |
| 32 | PD | | **LIKE** | 'F150%' | **OR** | | | | | | |
| 33 | PD | | **LIKE** | 'F151%' | **OR** | | | | | | |
| 34 | PD | | **LIKE** | 'F152%' | **OR** | | | | | | |
| 35 | PD | | **LIKE** | 'F153%' | **OR** | | | | | | |
| 36 | PD | | **LIKE** | 'F154%' | **OR** | | | | | | |
| 37 | PD | | **LIKE** | 'F155%' | **OR** | | | | | | |
| 38 | PD | | **LIKE** | 'F156%' | **OR** | | | | | | |
| 39 | PD | | **LIKE** | 'F157%' | **OR** | | | | | | |
| 40 | PD | | **LIKE** | 'F158%' | **OR** | | | | | | |
| 41 | PD | | **LIKE** | 'F159%' | **OR** | | | | | | |
| 42 | PD | | **LIKE** | 'F190%' | **OR** | | | | | | |
| 43 | PD | | **LIKE** | 'F191%' | **OR** | | | | | | |
| 44 | PD | | **LIKE** | 'F192%' | **OR** | | | | | | |
| 45 | PD | | **LIKE** | 'F193%' | **OR** | | | | | | |
| 46 | PD | | **LIKE** | 'F194%' | **OR** | | | | | | |
| 47 | PD | | **LIKE** | 'F195%' | **OR** | | | | | | |
| 48 | PD | | **LIKE** | 'F196%' | **OR** | | | | | | |
| 49 | PD | | **LIKE** | 'F197%' | **OR** | | | | | | |
| 50 | PD | | **LIKE** | 'F198%' | **OR** | | | | | | |
| 51 | PD | | **LIKE** | 'F199%' | **OR** | | | | | | |
| 52 | PD | | **LIKE** | 'F52%' | **OR** | | | | | | |
| 53 | PD | | **LIKE** | 'F55%' | **OR** | | | | | | |
| 54 | PD | | **LIKE** | 'G210%' | **OR** | | | | | | |
| 55 | PD | | **LIKE** | 'G211%' | **OR** | | | | | | |
| 56 | PD | | **LIKE** | 'G212%' | **OR** | | | | | | |
| 57 | PD | | **LIKE** | 'G240%' | **OR** | | | | | | |
| 58 | PD | | **LIKE** | 'G251%' | **OR** | | | | | | |
| 59 | PD | | **LIKE** | 'G254%' | **OR** | | | | | | |
| 60 | PD | | **LIKE** | 'G256%' | **OR** | | | | | | |
| 61 | PD | | **LIKE** | 'G444%' | **OR** | | | | | | |
| 62 | PD | | **LIKE** | 'G620%' | **OR** | | | | | | |
| 63 | PD | | **LIKE** | 'G720%' | **OR** | | | | | | |
| 64 | PD | | **LIKE** | 'H263%' | **OR** | | | | | | |
| 65 | PD | | **LIKE** | 'H406%' | **OR** | | | | | | |
| 66 | PD | | **LIKE** | 'H53%' | **OR** | | | | | | |
| 67 | PD | | **LIKE** | 'H910%' **OR** | | | | | | | |
| 68 | PD | | **LIKE** | 'I158%' **OR** | | | | | | | |
| 69 | PD | | **LIKE** | 'I159%' **OR** | | | | | | | |
| 70 | PD | | **LIKE** | 'I427%' **OR** | | | | | | | |
| 71 | PD | | **LIKE** | 'I952%' **OR** | | | | | | | |
| 72 | PD | | **LIKE** | 'J451%' **OR** | | | | | | | |
| 73 | PD | | **LIKE** | 'J702%' **OR** | | | | | | | |
| 74 | | | PD | **LIKE** | 'J703%' | | **OR** |  |  |  |  |
| 75 | | | PD | **LIKE** | 'J704%' | | **OR** |  |  |  |  |
| 76 | | | PD | **LIKE** | 'K250%' | | **OR** |  |  |  |  |
| 77 | | | PD | **LIKE** | 'K251%' | | **OR** |  |  |  |  |
| 78 | | | PD | **LIKE** | 'K252%' | | **OR** |  |  |  |  |
| 79 | | | PD | **LIKE** | 'K253%' | | **OR** |  |  |  |  |
| 80 | | | PD | **LIKE** | 'K254%' | | **OR** |  |  |  |  |
| 81 | | | PD | **LIKE** | 'K255%' | | **OR** |  |  |  |  |
| 82 | | | PD | **LIKE** | 'K256%' | | **OR** |  |  |  |  |
| 83 | | | PD | **LIKE** | 'K257%' | | **OR** |  |  |  |  |
| 84 | | | PD | **LIKE** | 'K259%' | | **OR** |  |  |  |  |
| 85 | | | PD | **LIKE** | 'K260%' | | **OR** |  |  |  |  |
| 86 | | | PD | **LIKE** | 'K261%' | | **OR** |  |  |  |  |
| 87 | | | PD | **LIKE** | 'K262%' | | **OR** |  |  |  |  |
| 88 | | | PD | **LIKE** | 'K263%' | | **OR** |  |  |  |  |
| 89 | | | PD | **LIKE** | 'K264%' | | **OR** |  |  |  |  |
| 90 | | | PD | **LIKE** | 'K265%' | | **OR** |  |  |  |  |
| 91 | | | PD | **LIKE** | 'K266%' | | **OR** |  |  |  |  |
| 92 | | | PD | **LIKE** | 'K267%' | | **OR** |  |  |  |  |
| 93 | | | PD | **LIKE** | 'K269%' | | **OR** |  |  |  |  |
| 94 | | | PD | **LIKE** | 'K270%' | | **OR** |  |  |  |  |
| 95 | | | PD | **LIKE** | 'K271%' | | **OR** |  |  |  |  |
| 96 | | | PD | **LIKE** | 'K272%' | | **OR** |  |  |  |  |
| 97 | | | PD | **LIKE** | 'K273%' | | **OR** |  |  |  |  |
| 98 | | | PD | **LIKE** | 'K274%' | | **OR** |  |  |  |  |
| 99 | | | PD | **LIKE** | 'K275%' | | **OR** |  |  |  |  |
| 100 | | | PD | **LIKE** | 'K276%' | | **OR** |  |  |  |  |
| 101 | | | PD | **LIKE** | 'K277%' | | **OR** |  |  |  |  |
| 102 | | | PD | **LIKE** | 'K279%' | | **OR** |  |  |  |  |
| 103 | | | PD | **LIKE** | 'K28%' **OR** | | |  |  |  |  |
| 104 | | | PD | **LIKE** | 'K290%' | | **OR** |  |  |  |  |
| 105 | | | PD | **LIKE** | 'K521%' | | **OR** |  |  |  |  |
| 106 | | | PD | **LIKE** | 'K529%' | | **OR** |  |  |  |  |
| 107 | | | PD | **LIKE** | 'K710%' | | **OR** |  |  |  |  |
| 108 | | | PD | **LIKE** | 'K711%' | | **OR** |  |  |  |  |
| 109 | | | PD | **LIKE** | 'K712%' | | **OR** |  |  |  |  |
| 110 | | | PD | **LIKE** | 'K713%' | | **OR** |  |  |  |  |
| 111 | | | PD | **LIKE** | 'K714%' | | **OR** |  |  |  |  |
| 112 | | | PD | **LIKE** | 'K715%' | | **OR** |  |  |  |  |
| 113 | | | PD | **LIKE** | 'K716%' | | **OR** |  |  |  |  |
| 114 | | | PD | **LIKE** | 'K717%' | | **OR** |  |  |  |  |
| 115 | | | PD | **LIKE** | 'K718%' | | **OR** |  |  |  |  |
| 116 | | | PD | **LIKE** | 'K719%' | | **OR** |  |  |  |  |
| 117 | | | PD | **LIKE** | 'K853%' | | **OR** |  |  |  |  |
| 118 | | | PD | **LIKE** | 'L105%' | | **OR** |  |  |  |  |
| 119 | | | PD | **LIKE** | 'L233%' | | **OR** |  |  |  |  |
| 120 | | | PD | **LIKE** | 'L244%' | | **OR** |  |  |  |  |
| 121 | | | PD | **LIKE** | 'L251%' | | **OR** |  |  |  |  |
| 122 | | | PD | **LIKE** | 'L27%' **OR** | | |  |  |  |  |
| 123 | | | PD | **LIKE** | 'L290%' **OR** | | |  |  |  |  |
| 124 | | | PD | **LIKE** | 'L291%' **OR** | | |  |  |  |  |
| 125 | | | PD | **LIKE** | 'L292%' **OR** | | |  |  |  |  |
| 126 | | | PD | **LIKE** | 'L293%' **OR** | | |  |  |  |  |
| 127 | | | PD | **LIKE** | 'L298%' **OR** | | |  |  |  |  |
| 128 | | | PD | **LIKE** | 'L299%' **OR** | | |  |  |  |  |
| 129 | | | PD | **LIKE** | 'L432%' **OR** | | |  |  |  |  |
| 130 | | | PD | **LIKE** | 'L500%' **OR** | | |  |  |  |  |
| 131 | | | PD | **LIKE** | 'L51%' **OR** | | |  |  |  |  |
| 132 | | | PD | **LIKE** | 'L560%' | | **OR** |  |  |  |  |
| 133 | | | PD | **LIKE** | 'L561%' | | **OR** |  |  |  |  |
| 134 | | | PD | **LIKE** | 'L562%' | | **OR** |  |  |  |  |
| 135 | | | PD | **LIKE** | 'L640%' | | **OR** |  |  |  |  |
| 136 | | | PD | **LIKE** | 'M022%' | | **OR** |  |  |  |  |
| 137 | | | PD | **LIKE** | 'M102%' | | **OR** |  |  |  |  |
| 138 | | | PD | **LIKE** | 'M320%' | | **OR** |  |  |  |  |
| 139 | | | PD | **LIKE** | 'M342%' | | **OR** |  |  |  |  |
| 140 | | | PD | **LIKE** | 'M804%' | | **OR** |  |  |  |  |
| 141 | | | PD | **LIKE** | 'M814%' | | **OR** |  |  |  |  |
| 142 | | | PD | **LIKE** | 'M835%' | | **OR** |  |  |  |  |
| 143 | | | PD | **LIKE** | 'M871%' | | **OR** |  |  |  |  |
| 144 | | | PD | **LIKE** | 'N140%' | | **OR** |  |  |  |  |
| 145 | | | PD | **LIKE** | 'N141%' | | **OR** |  |  |  |  |
| 146 | | | PD | **LIKE** | 'N142%' | | **OR** |  |  |  |  |
| 147 | | | PD | **LIKE** | 'N143%' **OR** | | |  |  |  |  |
| 148 | | | PD | **LIKE** | 'N144%' **OR** | | |  |  |  |  |
| 149 | | | PD | **LIKE** | 'N17%' | **OR** | |  |  |  |  |
| 150 | | | PD | **LIKE** | 'N18%' | **OR** | |  |  |  |  |
| 151 | | | PD | **LIKE** | 'N19%' | **OR** | |  |  |  |  |
| 152 | | | PD | **LIKE** | 'N990%' | **OR** | |  |  |  |  |
| 153 | | | PD | **LIKE** | 'O266%' | **OR** | |  |  |  |  |
| 154 | | | PD | **LIKE** | 'O355%' | **OR** | |  |  |  |  |
| 155 | | | PD | **LIKE** | 'O742%' | **OR** | |  |  |  |  |
| 156 | | | PD | **LIKE** | 'O743%' | **OR** | |  |  |  |  |
| 157 | | | PD | **LIKE** | 'O744%' | **OR** | |  |  |  |  |
| 158 | | | PD | **LIKE** | 'O746%' | **OR** | |  |  |  |  |
| 159 | | | PD | **LIKE** | 'P040%' | **OR** | |  |  |  |  |
| 160 | | | PD | **LIKE** | 'P041%' | **OR** | |  |  |  |  |
| 161 | | | PD | **LIKE** | 'P044%' | **OR** | |  |  |  |  |
| 162 | | | PD | **LIKE** | 'P584%' | **OR** | |  |  |  |  |
| 163 | | | PD | **LIKE** | 'P93%' | **OR** | |  |  |  |  |
| 164 | | | PD | **LIKE** | 'P961%' **OR** | | |  |  |  |  |
| 165 | | | PD | **LIKE** | 'P962%' **OR** | | |  |  |  |  |
| 166 | | | PD | **LIKE** | 'Q861%' **OR** | | |  |  |  |  |
| 167 | | | PD | **LIKE** | 'Q862%' **OR** | | |  |  |  |  |
| 168 | | | PD | **LIKE** | 'R502%' **OR** | | |  |  |  |  |
| 169 | | | PD | **LIKE** | 'T36%' | **OR** | |  |  |  |  |
| 170 | | | PD | **LIKE** | 'T37%' | **OR** | |  |  |  |  |
| 171 | | | PD | **LIKE** | 'T38%' | **OR** | |  |  |  |  |
| 172 | | | PD | **LIKE** | 'T39%' | **OR** | |  |  |  |  |
| 173 | | | PD | **LIKE** | 'T4%' **OR** | | |  |  |  |  |
| 174 | | | PD | **LIKE** | 'T5%' **OR** | | |  |  |  |  |
| 175 | | | PD | **LIKE** | 'T60%' | **OR** | |  |  |  |  |
| 176 | | | PD | **LIKE** | 'T61%' | **OR** | |  |  |  |  |
| 177 | | | PD | **LIKE** | 'T62%' | **OR** | |  |  |  |  |
| 178 | | | PD | **LIKE** | 'T63%' | **OR** | |  |  |  |  |
| 179 | | | PD | **LIKE** | 'T64%' | **OR** | |  |  |  |  |
| 180 | | | PD | **LIKE** | 'T65%' | **OR** | |  |  |  |  |
| 181 | | | PD | **LIKE** | 'T78%' | **OR** | |  |  |  |  |
| 182 | | | PD | **LIKE** | 'T801%' | **OR** | |  |  |  |  |
| 183 | | | PD | **LIKE** | 'T802%' | **OR** | |  |  |  |  |
| 184 | | | PD | **LIKE** | 'T803%' | **OR** | |  |  |  |  |
| 185 | | | PD | **LIKE** | 'T804%' | **OR** | |  |  |  |  |
| 186 | | | PD | **LIKE** | 'T805%' | **OR** | |  |  |  |  |
| 187 | | | PD | **LIKE** | 'T806%' | **OR** | |  |  |  |  |
| 188 | | | PD | **LIKE** | 'T808%' | **OR** | |  |  |  |  |
| 189 | | | PD | **LIKE** | 'T809%' | **OR** | |  |  |  |  |
| 190 | | | PD | **LIKE** | 'T880%' | **OR** | |  |  |  |  |
| 191 | | | PD | **LIKE** | 'T881%' | **OR** | |  |  |  |  |
| 192 | | | PD | **LIKE** | 'T883%' | **OR** | |  |  |  |  |
| 193 | | | PD | **LIKE** | 'T885%' | **OR** | |  |  |  |  |
| 194 | | | PD | **LIKE** | 'T886%' | **OR** | |  |  |  |  |
| 195 | | | PD | **LIKE** | 'T887%' | **OR** | |  |  |  |  |
| 196 | | | PD | **LIKE** | 'T96%' | **OR** | |  |  |  |  |
| 197 | | | PD | **LIKE** | 'X40%' | **OR** | |  |  |  |  |
| 198 | | | PD | **LIKE** | 'X41%' | **OR** | |  |  |  |  |
| 199 | | | PD | **LIKE** | 'X42%' | **OR** | |  |  |  |  |
| 200 | | | PD | **LIKE** | 'X43%' | **OR** | |  |  |  |  |
| 201 | | | PD | **LIKE** | 'X44%' | **OR** | |  |  |  |  |
| 202 | | | PD | **LIKE** | 'X455%' **OR** | | |  |  |  |  |
| 203 | | | PD | **LIKE** | 'Y10%' | **OR** | |  |  |  |  |
| 204 | | | PD | **LIKE** | 'Y40%' | **OR** | |  |  |  |  |
| 205 | | | PD | **LIKE** | 'Y41%' | **OR** | |  |  |  |  |
| 206 | | | PD | **LIKE** | 'Y42%' | **OR** | |  |  |  |  |
| 207 | | | PD | **LIKE** | 'Y427%' **OR** | | |  |  |  |  |
| 208 | | | PD | **LIKE** | 'Y43%' | **OR** | |  |  |  |  |
| 209 | | | PD | **LIKE** | 'Y44%' | **OR** | |  |  |  |  |
| 210 | | | PD | **LIKE** | 'Y45%' | **OR** | |  |  |  |  |
| 211 | | | PD | **LIKE** | 'Y46%' | **OR** | |  |  |  |  |
| 212 | | | PD | **LIKE** | 'Y47%' | **OR** | |  |  |  |  |
| 213 | | | PD | **LIKE** | 'Y48%' | **OR** | |  |  |  |  |
| 214 | | | PD | **LIKE** | 'Y49%' | **OR** | |  |  |  |  |
| 215 | | | PD | **LIKE** | 'Y50%' | **OR** | |  |  |  |  |
| 216 | | | PD | **LIKE** | 'Y51%' | **OR** | |  |  |  |  |
| 217 | | | PD | **LIKE** | 'Y52%' | **OR** | |  |  |  |  |
| 218 | | | PD | **LIKE** | 'Y53%' | **OR** | |  |  |  |  |
| 219 | | | PD | **LIKE** | 'Y54%' | **OR** | |  |  |  |  |

1. PD **LIKE** 'Y55%' **OR**
2. PD **LIKE** 'Y56%' **OR**
3. PD **LIKE** 'Y57%' **OR**
4. PD **LIKE** 'Y58%' **OR**
5. PD **LIKE** 'Y59%' **OR**
6. PD **LIKE** 'Y63%' **OR**
7. PD **LIKE** 'Y651%' **OR**
8. PD **LIKE** 'Y66%' **OR**
9. PD **LIKE** 'Y69%' **OR**
10. PD **LIKE** 'Y880%' **OR**
11. PD **LIKE** 'Z036%'

231 **)**

# **OR**

1. **(**
2. -- Ancillary information path (4 prefixes)
3. ANC **LIKE** 'Y4%' **OR**
4. ANC **LIKE** 'Y5%' **OR**
5. ANC **LIKE** 'X499%' **OR**
6. ANC **LIKE** 'X849%'

239 **)**

1. **OR**
2. **(**
3. -- First secondary diagnosis path (14 prefixes)
4. SD1 **LIKE** 'Y4%' **OR**
5. SD1 **LIKE** 'Y5%' **OR**
6. SD1 **LIKE** 'T36%' **OR**
7. SD1 **LIKE** 'T37%' **OR**
8. SD1 **LIKE** 'T38%' **OR**
9. SD1 **LIKE** 'T39%' **OR**
10. SD1 **LIKE** 'T4%' **OR**
11. SD1 **LIKE** 'T5%' **OR**
12. SD1 **LIKE** 'T60%' **OR**
13. SD1 **LIKE** 'T61%' **OR**
14. SD1 **LIKE** 'T62%' **OR**
15. SD1 **LIKE** 'T63%' **OR**
16. SD1 **LIKE** 'T64%' **OR**
17. SD1 **LIKE** 'T65%'

257 **);**
